# Not All Particles Are Equal: The Selective Enrichment of Particle-Associated Bacteria from the Mediterranean Sea

**DOI:** 10.3389/fmicb.2016.00996

**Published:** 2016-06-22

**Authors:** Mario López-Pérez, Nikole E. Kimes, Jose M. Haro-Moreno, Francisco Rodriguez-Valera

**Affiliations:** Evolutionary Genomics Group, División de Microbiología, Universidad Miguel HernándezAlicante, Spain

**Keywords:** metagenomics, microbial diversity, particle-attached bacteria, free-living bacteria, phages, Mediterranean Sea, particle-enrichments

## Abstract

We have used two metagenomic approaches, direct sequencing of natural samples and sequencing after enrichment, to characterize communities of prokaryotes associated to particles. In the first approximation, different size filters (0.22 and 5 μm) were used to identify prokaryotic microbes of free-living and particle-attached bacterial communities in the Mediterranean water column. A subtractive metagenomic approach was used to characterize the dominant microbial groups in the large size fraction that were not present in the free-living one. They belonged mainly to Actinobacteria, Planctomycetes, Flavobacteria and Proteobacteria. In addition, marine microbial communities enriched by incubation with different kinds of particulate material have been studied by metagenomic assembly. Different particle kinds (diatomaceous earth, sand, chitin and cellulose) were colonized by very different communities of bacteria belonging to *Roseobacter, Vibrio, Bacteriovorax*, and *Lacinutrix* that were distant relatives of genomes already described from marine habitats. Besides, using assembly from deep metagenomic sequencing from the particle-specific enrichments we were able to determine a total of 20 groups of contigs (eight of them with >50% completeness) and reconstruct *de novo* five new genomes of novel species within marine clades (>79% completeness and <1.8% contamination). We also describe for the first time the genome of a marine Rhizobiales phage that seems to infect a broad range of Alphaproteobacteria and live in habitats as diverse as soil, marine sediment and water column. The metagenomic recruitment of the communities found by direct sequencing of the large size filter and by enrichment had nearly no overlap. These results indicate that these reconstructed genomes are part of the rare biosphere which exists at nominal levels under natural conditions.

## Introduction

Characterizing the true diversity of microbes in nature is a daunting task, yet great advances have been achieved through the use of high throughput sequencing (Sogin et al., 2006; Allen et al., 2012; Smith et al., 2013; Dupont et al., 2015). Next generation sequencing (NGS), in particular, has been extremely informative because it permits a glimpse into microbial diversity with very little bias and with an in depth view of the microbes that only the complete genome can provide (Temperton and Giovannoni, 2012). As a result, studying natural communities by direct sequencing has become common and has provided new insights into the diversity of microbes in many environments (Qin et al., 2010; Mackelprang et al., 2011; Fierer et al., 2012; DeLong, 2013). The potential for combining sequencing based studies (metagenomics) with that of culture based studies (enrichments), however, remains relatively unexploited. This combined approach provides a powerful tool to answer many biological queries, including the role of particulate matter in shaping marine microbial diversity, as we have done here.

An exciting transformation has occurred in our understanding of the oceans' influence on nutrient cycling over the past two decades, and long gone are the days when marine microorganisms were largely discounted as contributors to this process (Azam, 1998). Instead, we now recognize the oceans' microbial communities as important regulators of major oceanic biogeochemical cycles, capable of broad impacts (Jiao and Azam, 2011). Originally thought to simply assimilate and respire dissolved organic material (DOM), current evidence shows that many marine microorganisms actively sense, seek and transform particulate organic matter (POM) through various processes (Azam and Malfatti, 2007). This is an important contribution to ocean ecosystems that are largely oligotrophic environments containing patches of POM in the form of clustered and sinking particles, including detritus and marine snow (Azam and Long, 2001; Kiørboe and Jackson, 2001; Azam and Malfatti, 2007). Furthermore, genomic studies over the past decade have provided enlightening insights into the many mechanisms utilized by specific marine microbes in ocean ecosystem functioning (Azam and Worden, 2004; Azam and Malfatti, 2007; Allen et al., 2013).

Not all marine microorganisms are necessarily capable of sensing POM and subsequently moving to the source and transforming it into dissolved nutrients. Rather it has been proposed that there are two distinct communities in marine ecosystems: the particle-associated fraction, which is capable of colonizing POM and producing DOM, and the free-living fraction that thrives on the DOM released by the particle colonizers (Azam and Long, 2001; Kiørboe and Jackson, 2001; Allen et al., 2013). The greatest emphasis of marine microbial research has previously been on the free-living microorganisms (DeLong et al., 2006; Rusch et al., 2007; Dinsdale et al., 2008; Ghai et al., 2010), in large part due to the fact that many of the most abundant marine bacteria, e.g., *Pelagibacter ubique* (Giovannoni et al., 2005) and *Prochlorococcus* spp. (Rocap et al., 2003), are thought to be free-living. There is a large amount of evidence, however, providing support for the existence of particle-associated (PA) communities distinct from their free-living (FL) cohorts (Allen et al., 2013; Rieck et al., 2015). To date, only three direct sequencing metagenomic studies have been published, in which the differences in microbial community composition between presumably FL and PA communities based on size fractions. All three have focused on microbial communities in the Pacific Ocean, studying the coastal and open ocean waters off the Californian coast (Allen et al., 2012), the Columbia River coastal margin in the Pacific Northwest (Smith et al., 2013), and the oxygen minimum zone off the Chilean coast (Ganesh et al., 2014). These studies revealed a distinct taxonomic classification represented by an increased association with eukaryotes and viruses in the larger fractions, as well as, differences in functional classification with an increase in gene diversity associated with particle-associated fractions. These studies and also earlier studies based on 16S rRNA gene sequencing (DeLong et al., 1993; Crump et al., 1999) showed that the compositions of the PA communities were related to members of the Bacteroidetes (considered to be specialists in the degradation of high molecular weight organic matter), Plactomycetes and Proteobacteria (Gamma and Deltaproteobacteria). Recently, a combination of metagenome and metatrancriptome methodologies was used to analyze carbon and nutrient flux through free-living and particle-associated microbes of the Amazon Plume (Satinsky et al., 2014).

In the study presented here, we combine direct metagenomic sequencing with culture-based enrichments to explore the role of particles in shaping microbial diversity in the Mediterranean Sea. To initially characterize the size-fractionated microbial communities of the Mediterranean, we sequenced and analyzed two large metagenomic datasets of small (0.22–5.0 μm) and large (5.0–20.0 nμm) filter fractions theoretically representing the FL and PA communities, respectively. We also investigated the selective impact of different particles on community composition using metagenomic sequencing of particle-specific enrichments. Our results confirm that the Mediterranean FL and PA microbial communities are distinct. Moreover, we show that different particle chemical composition and structure can drive the enrichment of distinct and specific particle-associated community structures. In addition, using assembly from deep metagenomic sequencing from the particle-specific enrichments, we describe several nearly complete genomes most of them belonging to new species. These reconstructed genomes are part of the rare biosphere, which exists at nominal levels under natural conditions.

## Materials and methods

### Sample collection

For the free-living (FL, 0.22–5.0 μm fraction) and particle-associated (PA, 5.0–20.0 μm fraction) metagenomes, 200 L of Mediterranean seawater was collected off the coast of Alicante, Spain (38°4′6.64″N/0°13′55.18″W) on September 6th 2013 and January 27th 2015 from 55 and 20 m depth, respectively (16°C). The seawater was sequentially filtered using 20, 5, and 0.22 μm filters (GCWP2932A, Durapore, Millipore, Billerica, MA, USA), and the filters were immediately frozen at −20°C until processed for DNA extraction. For the particle-enriched (PE) metagenomes, seawater was also collected off the coast of Alicante, Spain (38°16′42″N/00°14′39″W) on May 20th 2013.

### Particle-enrichments

In the laboratory, 480 mL of seawater from 55 m depth was added to each of five flasks (2L) containing 50 g of one of the following: 1. seawater from the above sampling—control (SW), 2. Sand (SWsnd, Sigma 274739), 3. Diatomaceous Earth (SWde, Sigma D5509), 4. Cellulose (SWcel, Sigma S5504), 5. Chitin (SWchi, Sigma C7170) autoclaved and dissolved in 20 mL. Low levels of pyruvate (0.1% final concentration) and yeast extract (0.01% final concentration) were also added to each sample, which were then incubated for 2 weeks at 15°C with 12 h light/dark cycles with constant stirring. In order to collect particle-associated microorganism after these 2 weeks of incubation each particle-enriched culture (500 ml) was filtered through a 20 μm filter, allowing sea water to flow through and retaining only the particles and any bacteria attached to them. This was followed by 3 washes, in which 300 mL of sterile sea water was added to the particles. It should be noted that the control sample (SW), which did not contain any particles, was not subjected to any treatment until the end of the 5 weeks incubation. The whole sample was spun at 13,500 rpm for 30 min to pellet the microbial cells. A schematic representation of the enrichments experimental design is shown in Figure S1.

### DNA extractions and metagenomic sequencing

Community DNA extractions were performed for two FL and PA fractions (summer and winter), using the 0.22 and 5 μm filters, respectively. For the PE metagenomes, community DNA extractions were performed using either pelleted cells (SW) or rinsed and filtered particles (SWsnd, SWde, SWcel, and SWchi). In all cases, 5 mL of lysis buffer (40 mM EDTA, 50 mM Tris pH = 8.3, and 0.75M Sucrose) were added to the biomass (filters for FL and PA, cell pellet for SW and 5 mL of particles in all other samples) and incubated at 37°C for 45 min with 50 μL Lysozyme [100 mg/mL]. Nucleic acids were extracted with phenol/chloroform/isoamyl alcohol and chloroform/isoamyl alcohol and DNA integrity was checked by agarose gel electrophoresis. The metagenomes were sequenced using the Illumina Hi-Seq 2000 (100-bp paired-end read) sequencing platform by GATC Biotech (Konstanz, Germany). The IDBA-UD *de novo* assembler (Peng et al., 2012) was used to assemble all datasets using a Kmer range (*K* = 70–100, steps of 10). Gene predictions on the assembled contigs were performed using Prodigal (Hyatt et al., 2010), and tRNAs were predicted using tRNAscan-SE (Lowe and Eddy, 1997). Ribosomal rRNA genes were identified using ssu-align (Nawrocki, 2009) and meta_rna (Huang et al., 2009). Predicted proteins extracted from the contigs were assigned functional annotation using USEARCH (Edgar, 2010) against COG (Tatusov et al., 2001), TIGRFAM (Haft et al., 2001) and NCBI nr databases. TIGRFAM and COG assignments were made using an evalue of <1e-5, and >50% query coverage and >30% identity for non-redundant NCBI. Particle-associated metagenome were also annotated using the RAST server (Aziz et al., 2008). The completeness and contamination of each reconstructed genome were estimated using CheckM with default settings (Parks et al., 2015). The completeness was estimated by identification of lineage-specific single-copy marker genes and contamination was evaluated from the number of multi-copy marker genes.

### Phylogenetic and functional classifications

Phylogenetic classification of the metagenomic data was performed using three different methods. In the first, ribosomal RNA (rRNA) sequences were used as queries for BLASTN searches against the entire RDP database and classified into a high level taxon if the sequence identity was ≥80% and the alignment length was ≥90 bp. For the second method MetaPhlAn version (v2.0) was used to study the microbial community (Segata et al., 2012) from the total reads. This program maps reads to a set of selected marker sequences that are unique for each organism in the database. In addition, we used only the annotation of large contigs (> 10 Kb) to make class-level and organism-level assignments. Genome trees were made for phylogenetic comparisons using conserved protein sequences among the microorganisms used in a given comparison. Conserved proteins were identified for each genome and the reference genomes using the COG database (Tatusov et al., 2001). COG assignments were made using the HMMER software package version 2.0 (Finn et al., 2011) with an evalue of <1e-5, >80% query coverage and >30% identity. The common conserved proteins were identified and concatenated. The resulting concatenated protein sequences were then aligned using Kalign (Lassmann and Sonnhammer, 2005), trimmed using trimAL (Capella-Gutiérrez et al., 2009). A maximum likelihood tree was constructed using FastTree2 (Price et al., 2010) using a JTT+CAT model, a gamma approximation and with 100 bootstrap replicates.

### Recruitment analysis

The assembled contig groups for each of our PE metagenomes, which were identified at the genus/species level, were compared to a number of metagenomic datasets from seawater to evaluate the coverage of each contig group in natural samples. Recruitments were performed using BLASTN (Altschul et al., 1997) and a hit was considered only when it was at least 50 nucleotides long, with a %identity of >95% and with an *e*-value ≤ 1e-5. The positive results were calculated and reported as reads per Kb per Gb of data (RPKG) that provide a normalized value comparable across various metagenomes (Mizuno et al., 2013).

### Data availability

The metagenomic data and assembled genome sequences have been submitted to NCBI under the Bioproject identifier PRJNA257723 for the PA and FL metagenomes and PRJNA318731 for PE metagenomes. FL-Summer and PA-Summer metagenomes have been submitted as MedDCM-SEP2013 (BioSample SAMN02954150) and MedDCM-SEP2013-LF (BioSample SAMN02954152), respectively. FL-Winter and PA-Winter metagenomes have been submitted as MedWinter-JAN2015-20m (BioSample SAMN04874669) and MedWinter-JAN2015-20m-LF (BioSample SAMN04880534), respectively.

## Results and discussion

### Comparison of free-living (FL) and particle-associated (PA) metagenomes

In order to investigate the differences between microbial communities associated with the FL and PA fractions of pelagic seawater in the Mediterranean Sea, we first used size-based filter fractions to produce metagenomes. We compared four large (one Illumina lane) metagenomes sequenced from either the 0.22–5.0 μm (FL) or the 5.0–20.0 μm (PA) filter fractions of two separate samples. One sample was taken during the summer from the deep chlorophyll maximum (DCM), a zone of maximal phytoplankton concentration that generally occurs below the thermocline. In tropical waters this is a permanent feature, whereas in the Mediterranean and other temperate waters the DCM is a seasonal phenomenon (summer and part of spring and autumn). The second sample was collected during the winter mixing that occurs in January. However, the [Chl *a*] concentration (mg m^−3^) was only slightly higher during the summer sampling (0.92) compared to the winter sampling (0.75) but similar to the average [Chl *a*] observed at the DCM depth in different points in the southwestern Mediterranean Sea that reaches 0.88 mg m^−3^ (Lavigne et al., 2015). Approximately, 12 Gb of raw sequence were retrieved from each data set (Table S1). Figure S2 shows the GC% distribution plot of the individual reads in the data sets. Both FL samples and the PA from 2014 clearly show a peak at *ca*. 34% GC consistent with the GC content of the dominant microbe based on rRNA reads, *Ca*. Pelagibacter (%GC *ca*. 30). Interestingly, however, the summer PA fraction had a bimodal GC distribution (Figure S2) with maxima at 39 and 50%. Both PA metagenomes revealed much higher percentages of eukaryotic and viral sequences and lower percentages of bacteria and archaea based on both rRNA analysis and the MetaPhlAn analysis from the total reads (Figure S3). The ratio of eukaryotic to bacterial rRNA (18S:16S) was (0.63 and 0.32) for PA summer and winter respectively and (0.036 and 0.063) for FL. Viral DNA was similarly overrepresented in PA regardless of the season (Figure S3). Also the percentage of sequences with predicted proteins of known function was much lower in PA than in FL metagenomes (*ca*.16 and *ca*.40% respectively) independent of the season. These results are similar with previous observations in particles enriched metagenomes (Ganesh et al., 2014; Dupont et al., 2015; Fontanez et al., 2015) and can be largely attributed to the presence of more eukaryotic DNA in the PA fraction. The presence of phage genomes in metagenomes is due to the abundance in the population of cells undergoing the lytic cycle. During this phase a large amount of phage DNA accumulated within the cell and is retrieved with the cellular fraction (Mizuno et al., 2013). Therefore, higher relative abundance of phage reads in the particulate fraction can be interpreted as a reflection of higher infection rate in this cellular fraction (Allen et al., 2012; Ganesh et al., 2014; Dupont et al., 2015).

**Figure 1:**
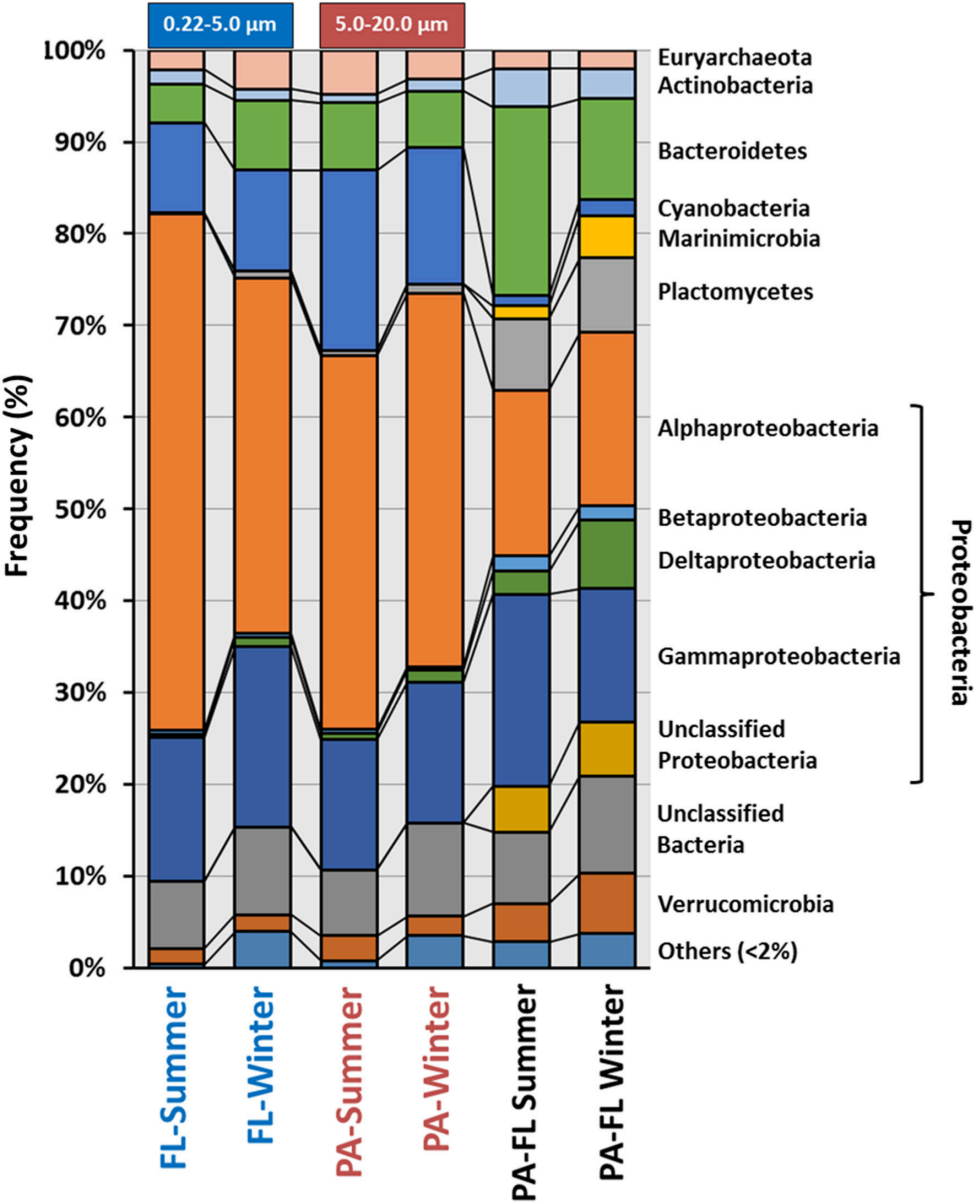
**Class-level composition of the domain Bacteria and Archaea based on rRNA (raw reads) among the four individual metagenomes and the subtractive bacterial community associated to the PA metagenomes**. For the subtraction, we used prokaryotic rRNA sequences that were found to be unique (i.e., meaning they exhibited >95% identity for over 90 bp to any sequence in the FL metagenomes) to the PA fraction. Free-living (FL); Particulate-associated (PA).

Based on rRNA data, at the level of major phylogenetic clades, the community structure of all four metagenomes was remarkably well conserved (Figure 1). In order to better characterize the bacterial community associated to the PA, we examined 16S rRNA reads that were unique to the PA metagenomes by removing sequences that exhibited >95% identity for over 90 bp to any sequence in the FL metagenomes. Around 10% of the 16S rRNA sequences (11.1% for summer PA and 8.7% for winter PA) were found to be unique to the PA fraction (Figure 1, Table S2). The most abundant groups among the total PA reads were Proteobacteria (Alpha and Gamma), Bacteroidetes and Cyanobacteria. Whereas, after the subtraction, Proteobacteria (Alpha, Delta, and Gamma), Bacteroidetes (Flavobacteria), and Planctomycetes (Planctomycetia) were significantly overrepresented, providing additional support that certain microorganisms within these classes are unique to the PA community (Figure 1) and consistent with several previous studies of particle-associated bacteria (DeLong et al., 1993; Allen et al., 2012; Ganesh et al., 2014). The percentage of Planctomycetia increased from less than 1% in the individual metagenomes to more than 7% in the subtraction in both comparisons (summer and winter), while the same is true for the Verrucomicrobia class Opitutae in the winter sample. Previous studies have demonstrated that marine bacteria belonging to the phylum Planctomycetes are more abundant in sinking marine aggregates (DeLong et al., 1993), in the biofilm community living on the surface of different marine species (Gray and Herwig, 1996; Longford et al., 2007; Bengtsson and Øvreås, 2010) and in particle-associated communities (Allgaier and Grossart, 2006). This data also revealed that the Cyanobacteria present in both fractions represented the same groups. Clogging of the PA filter could have been the reason for the retention of high amount of Cyanobacteria in the PA fraction (Crespo et al., 2013), another explanation is that mucous aggregates of cyanobacteria, previously demonstrated (Deng et al., 2015), hindered their passage through the 5 μm filter. Notably, unclassified bacteria were among the largest groups of unique rRNA reads (Figure 1, Table S2), even at higher taxonomic levels, suggesting that a significant portion of microorganisms specific to the PA fraction have yet to be characterized.

**Figure 2:**
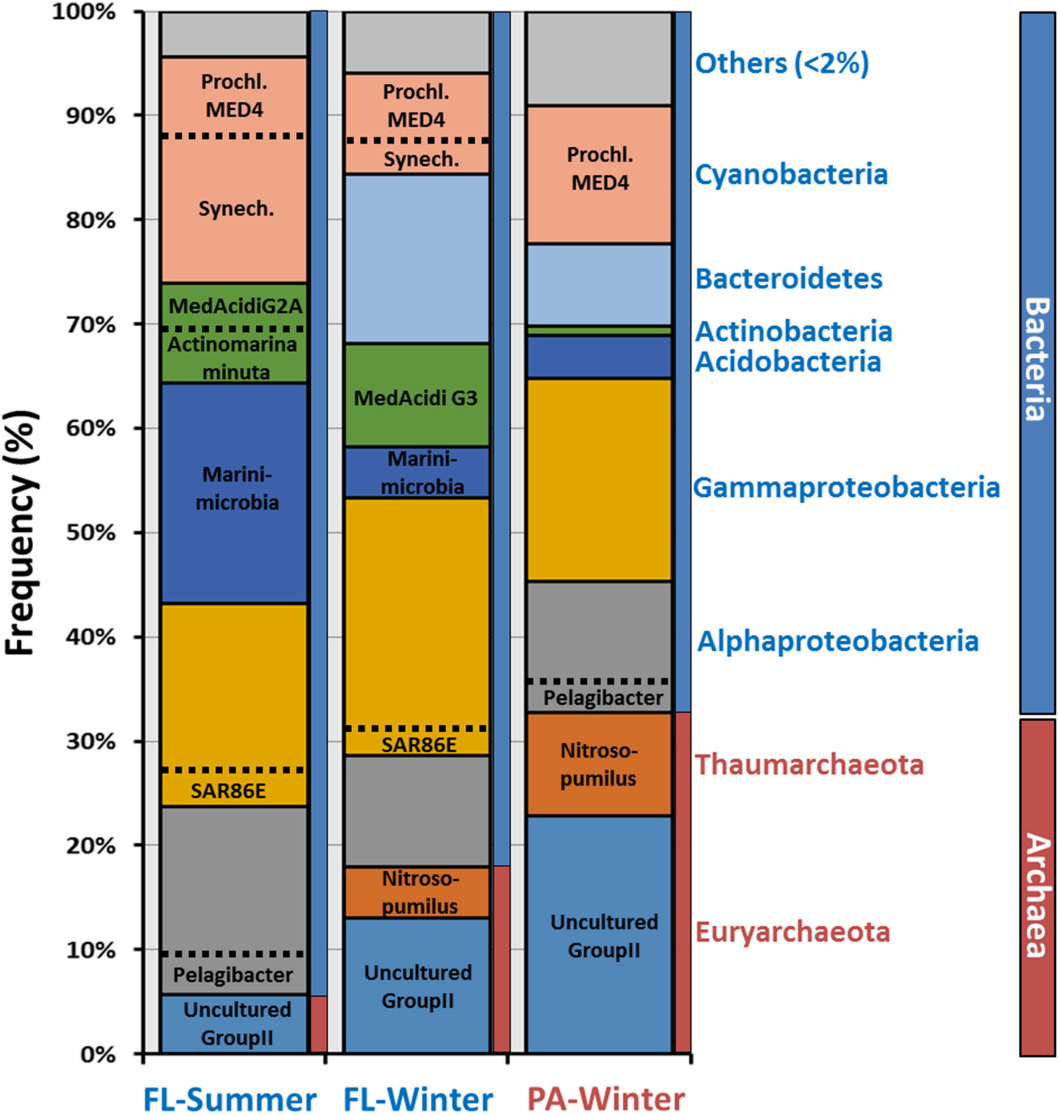
**Class-level phylogenetic classification of annotated contigs >10 Kb**. When it was possible, contigs were associated at the genus-level based on annotation. Prochl, *Prochlorococcus*; Synech, *Synechococcus.*

**Figure 3:**
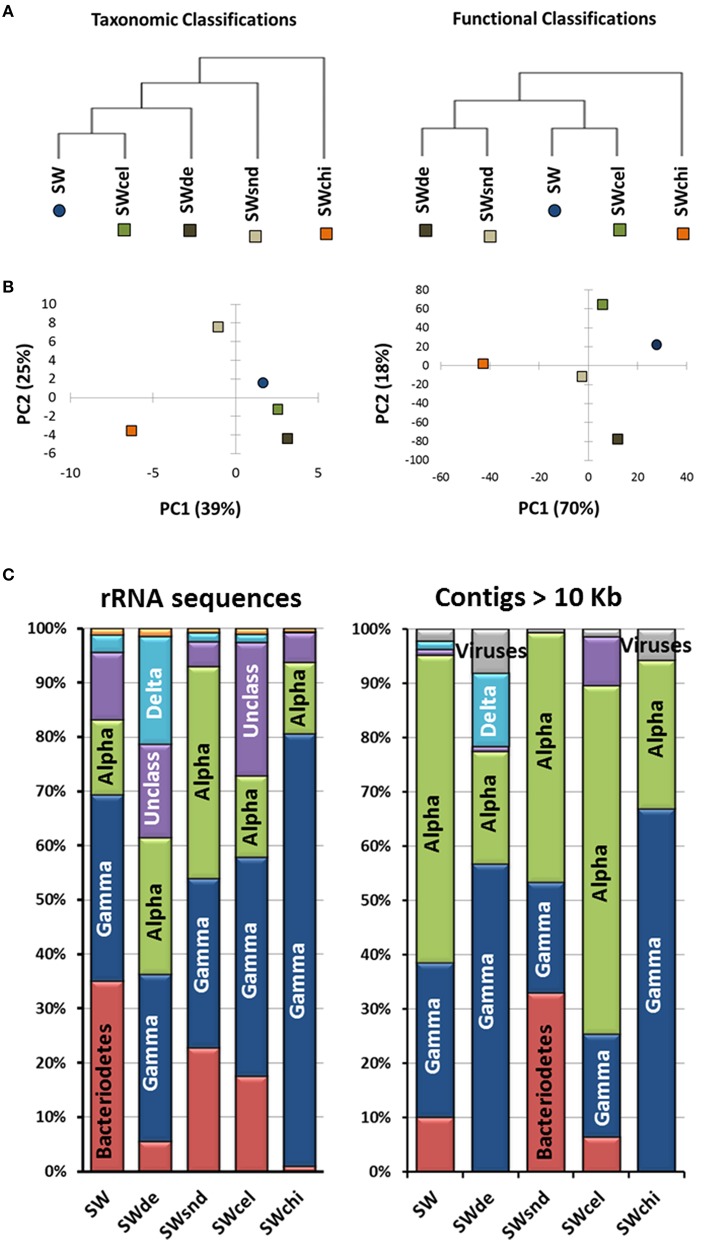
**Analyses of the particle-enrichment metagenomes. (A)** Metagenomes tree, **(B)** principle component analysis (PCA) analysis related to taxonomic and functional classifications, and **(C)** phylogenetic classification of both rRNA sequences (>100 bp) from the raw metagenomic reads and annotated contigs >10 Kb for the SW, (sea water-control); SWsnd, (Sand); SWde, (Diatomaceous Earth); SWcel, (Cellulose); and SWchi, (Chitin) metagenomes.

We performed *de novo* contig assembly using the complete set of raw reads to further analyze these data sets (Table S1). As is expected when there are large numbers of eukaryotic sequences and significant differences in GC content, we were unable to assemble large contigs from the PA summer metagenome. Nonetheless, we were able to assemble and compare the other three metagenomes. After assembly, contigs can be classified much more reliably using large genomic fragments (minimum 10 Kb lengths). However, it is important to emphasize that microbes are not assembled proportionally to their abundance, i.e., assembly is only a rough estimate of the relative abundance. Assembled contigs were assigned to each class based on the majority of genes giving highest similarities to genes in genomes present in NCBI nr database (Figure 2). Alphaproteobateria, Gammaproteobacteria, and Cyanobacteria were the most abundant groups in both fractions, consistent with the previously described rRNA classifications. A very remarkable difference found was the proportion of members of Archaea that increased from 5 to 18% (summer and winter FL) to 32% in the winter PA metagenome. In both, the FL and PA fractions Euryarchaeota, the majority of which were Group II Euryarchaeota, were the dominant class over Thaumarchaeota. Actinobacteria were more abundant in both FL fractions, reaching up to 10%, compared to the assembled PA contigs, in which it was practically absent (Figure 2). The summer FL metagenome contained mostly *Ca.* Actinobacteria minuta (Ghai et al., 2013) and MedAcidi-G2A (Mizuno et al., 2015), while MedAcidi-G3 was predominant in the winter FL. This data suggests that these Actinobacteria do not form aggregates and can pass the 5 μm filter. The majority of Gammaproteobacteria contigs were unclassified (Figure 3), suggesting the possibility of novel planktonic lineages. Cyanobacteria were more abundant in the summer FL fraction (21%) than in the winter FL and PA fraction (10 and 13%, respectively). Moreover, the proportion of the two most abundant Cyanobacteria genera (*Prochlorococcus* and *Synechococcus*) varied among the three different metagenomes (Figure 2). The winter PA was particularly enriched in the high-light-adapted *Prochlorococcus* MED4. Although, this was also the most abundant in the other winter sample, *Synechococcus* represented 30% of the Cyanobacteria assembled sequences in the FL fraction. The percentage of *Synechococcus* increased to 70% in the summer FL.

### Particle enrichments (PE) and metagenomic sequencing

After comparing the FL and PA metagenomes derived from different size fractions, we designed another approximation to analyze the community that live associated to particles using enrichment metagenomics (Figure S1). In these experiments Mediterranean seawater was enriched with a specific particulate matter. After 2 weeks of growth under ecologically relevant conditions, microbial DNA was extracted for metagenomic sequencing (Table S3). Four types of particulate material were used. Two of them, sand (SWsnd) and diatomaceous earth (SWde) are both inorganic forms of silicates. Sand particles are larger with flat surfaces, while SWde are smaller and with perforated surfaces (larger surface/volume ratios). The other two particulate materials used, cellulose (SWcel) and chitin (SWchi), are organic polymers that could act as an additional carbon source to the pyruvate supplied, and both are abundant in marine POM (Figure S4).

Comparison among the assembled contigs (>1 kb) of the PE metagenomes based on principal component analysis (PCA) related to taxonomic and functional classifications reveal three clusters. The sequences from the control (SW), devoid of added particles, and the SWcel enrichment share a higher level of similarity in both comparisons, while the two with inorganic forms of silicates (SWsnd and SWde) grouped together only at the functional level. The most distinct metagenome was the chitin enriched (SWchi), which always appeared as an outgroup (Figures 3A,B). We used the phylogenetic classification of both annotated contigs >10 Kb and rRNA sequences (>100 bp) from the raw metagenomic reads (Figure 3C) to identify the dominant microorganisms in each metagenome. Even at the class level, we observed distinct community structures with both approaches. Members of the class Alphaproteobacteria (mostly *Roseobacter* spp) were the best assembled microbes. SWchi and SWde enrichments were dominated by Gammaproteobacteria and a small proportion of Viruses contigs respectively. Using the larger assembled contigs (>10 Kb) for classification also allowed us to identify a number of organisms at the genus/species level, providing further evidence of variability between the particle enrichments. We have determined a total of 20 groups of contigs associated to a specific microbe (Table 1) from these enrichments, although only eight of these groups of contigs obtained were partial to near complete (50–100%) and <4% contamination (see Materials and Methods, Table S4). These groups of contigs revealed a number of previously undescribed species within the Rhizobiales, Rhodobacteraceae, Flavobacteraceae, Bdellovibrionaceae, and Vibrionaceae families based on average nucleotide identity (ANI) comparisons between the assembled contigs and their corresponding most closely related cultured relative (Table 1).

**Table 1:** **Microorganisms identified from contig (>10kb) assembly in particle-enrichment metagenomes and comparison to the reference genomes**.

			**Assembled Genome**			**Reference Genome**		**Genomes Comparison**	
**Metagenome**	**Putative Assembled genome**	**Class**	**Length of the contigs (Mb)**	**Number of contigs**	**GC content contigs (%)**	**Genome size (Mb)**	**GC content (%)**	**ANI (%)**	**Coverage (%)**
SW	*Roseobacter* sp. MED193	Alphaproteobacteria	3.08	125	57.6	4.67	57.5	89.1	73.2
SW	*Lacinutrix* sp. 5H-3-7-4	Bacteriodetes	3.04	22	30.7	3.29	30.8	93.2	83.3
SW	*Rhizobium* sp.	Alphaproteobacteria	0.37	28	59.7	4.24	62	78.6	24.1
SW	*Pseudophaeobacter arcticus*	Alphaproteobacteria	0.17	7	59.5	5.05	59.3	82.3	37.3
SW	Unclassified	Gammaproteobacteria	1.04	73	40.9	–	–	–	–
SWde	*Roseobacter* sp. MED193	Alphaproteobacteria	4.31	19	57.3	4.67	57.5	98	87.4
SWde	*Bacteriovorax* sp. DB6 IX	Deltaproteobacteria	3.73	15	36.1	3	37.6	72.3	47.6
SWde	*Pseudoalteromonas haloplanktis* TAC125	Gammaproteobacteria	0.12	10	40.1	3.85	40.1	82.2	42.9
SWde	Unclassified	Gammaproteobacteria	0.82	63	41.2	–	–	–	–
SWcel	*Roseobacter* sp. MED193	Alphaproteobacteria	2.82	141	57.3	4.67	57.5	97.7	72.7
SWcel	*Flavobacterium beibuense*	Bacteriodetes	2.52	22	33.8	3.8	37.7	72.1	43.5
SWcel	*Gracilibacteria bacterium* JGI-0000069-K10	Gracilibacteria	0.16	12	28.3	0.76	23	70.7	20
SWcel	uncultured bacterium gcode4	no rank	0.14	10	28.8	–	–	–	–
SWcel	Unclassified	Alphaproteobacteria	4.03	57	61.1	–	–	–	–
SWcel	Unclassified	Gammaproteobacteria	0.84	65	40.7	–	–	–	–
SWchi	*Roseobacter* sp. MED193	Alphaproteobacteria	2.78	18	59	4.67	57.5	82.8	64.7
SWchi	*Vibrio* sp. F10	Gammaproteobacteria	2.6	26	45.3	4.41	43.3	76.7	53.1
SWchi	*Pseudoalteromonas undina*	Gammaproteobacteria	0.16	17	39.4	4	39.9	95.4	42.7
SWchi	*Pseudophaeobacter arcticus*	Alphaproteobacteria	0.12	3	59.4	5.05	59.3	81.1	33
SWsnd	*Roseobacter* sp. MED193	Alphaproteobacteria	2.68	151	58	4.67	57.5	89.8	63.4
SWsnd	*Crocinitomix catalasitica*	Bacteriodetes	1.7	95	36.7	4.62	34.1	66.7	25
SWsnd	*Pseudoalteromonas undina*	Gammaproteobacteria	0.7	46	40.5	4	39.9	97.6	55.7
SWsnd	*Pseudophaeobacter arcticus*	Alphaproteobacteria	0.45	27	59.1	5.05	59.3	81.5	42.2
SWsnd	*Pseudoalteromonas haloplanktis* TAC125	Gammaproteobacteria	0.21	14	41	3.85	40.1	79.8	40.5
SWsnd	Unclassified	Alphaproteobacteria	3.59	105	56.3	–	–	–	–
SWsnd	Unclassified	Bacteriodetes	6.03	162	34	–	–	–	–

### The same but different, roseobacter

All of the PE metagenomes contained Rhodobacteraceae contigs that were identified as *Roseobacter* sp. (Table 1). All vs. all ANI comparisons that were performed using the four Roseobacter contig groups and the closest sequenced relative, *Roseobacter* sp. MED193, in order to determine the phylogenetic relationship between the enriched *Roseobacter* spp. (Figure S5A). The results indicate that they represent three distinct species with only the SWde and SWcel sharing a high enough ANI to be considered as the same species as the cultured relative *Roseobacter* sp. MED193 (SWde, 98% ANI with 88% coverage; SWcel, 97.6% ANI with 73% coverage). Furthermore, SWsnd and SWchi contigs can be considered borderline within the same species sharing an ANI of 94.8% (58% coverage), while the SW *Roseobacter* spp represent a new *Roseobacter* species (Figure S5A). Phylogenetic analysis using 42 conserved proteins provided additional evidence supporting the four distinct Rhodobacteraceae groups (Figure S5B). The results indicate that particle size was the most relevant parameter to determine the overall community similarity i.e., similarly sized particles had similar communities regardless of the particle composition. For example, SWsnd and SWchi (large particles) had similar *Roseobacter* species attached although they are chemically very different.

### Specific microorganism from particle enrichments metagenomes

One group of 28 contigs from the SW control were identified as a *Rhizobium* sp. (Table 1), with the most closely related species being the newly described Mediterranean isolate, *Pseudorhizobium pelagicum* R1-200B4^T^ (Kimes et al., 2015). This microbe represents the first marine member of the family *Rhizobiaceae*. The alignment of the SW-Rhizobium contigs with the genome sequence of this isolate (accession NCBI number NZ_JOKI00000000.1) shows both sequence similarity and synteny, while the genome recruitment of *Pseudorhizobium pelagicum* R1-200B4^T^ with the SW metagenomic dataset reveals full coverage of the genome (Figure S6A). A conserved protein tree provides further evidence that the SW contigs form a cluster with both *Pseudorhizobium pelagicum* R1-200B4^T^ and *Rhizobium* sp. NT26 (Figure S6B). The second group of contigs detected in SW, a Flavobacteraceae group, was identified as a *Lacinutrix* sp., most closely resembling *Lacinutrix* sp 5H-3-7-4. This member of the family Flavobacteriaceae was isolated from subseafloor sediments using a mixture of cellulose, xylan, and chitin as the sole carbon sources in enrichment cultures (Klippel et al., 2011). The ANI (93% with 83% coverage) between the SW contigs and *Lacinutrix* sp 5H-3-7-4 genome indicated that, although potentially a different species, both are closely related. Moreover, the genome completeness based on CheckM was 99.34% with 0% of contaminations and this microbe recruited up to 34% of all reads belonging to the SW metagenome (Table S4). Alignment between contigs and the reference genome indicated that synteny was very well preserved except for the presence of some genomic islands. Although, most are associated with cell wall synthesis, we have found several glycoside hydrolases, such as laminarinase and alpha-amylase that could facilitate the use of several natural substrates as sources of carbon and energy. The SWcel metagenome contained only one group of Flavobacteraceae contigs (*n* = 22). However, unlike the SW contigs the SWcel were classified as a *Flavobacteria* sp. (Table 1). The most closely related microorganism identified, *Flavobacterium beibuense* F44-8^T^, clearly represented a different species (72% ANI with 44% coverage). This microbe was isolated from a crude-oil-degrading consortium, enriched from marine sediment (Fu et al., 2011). In total, the 23 Flavobacteria contigs, ranging between 136 and 12 Kb, represented more than one half (2.5 Mb) of the expected genome size (3.8 Mb). The genome was estimated to be 88.7% complete and with minimal contamination (Table S4). Recruitment of identical reads with the reference metagenome (SWcel) showed that 10.7% of the reads belonged to this *Flavobacterium beibuense*-like contigs. Their GC content was 33.8%, which is lower than that of *Flavobacterium beibuense* F44-8^T^ (37.7%). The contigs contain a cluster of 5 *crt* genes involved in carotenoid biosynthesis and genes associated with gliding motility, all typical characteristics of members of the class Flavobacteriia. No cellulases could be detected neither in the genome nor in the reference genome and it is possible that this specific microbe is only using the particles as physical support. Interestingly, we have found a 30 Kb putative prophage inserted in the genome with a significant gene similarity and synteny to another prophage inserted in the same position in a soil Bacteroidetes, *Flavobacterium* sp. CF136, isolated from the *Populus deltoids* rhizosphere (Brown et al., 2012). Based on ANI identity both strains represented different species (70% ANI with only 24% coverage).

Diatomaceous earth is made up of silicate remains of fossilized diatoms and (like in the case of sand) provides no organic carbon. The SWde metagenome contained a group of Deltaproteobacteria contigs identified as being most closely related to *Bacteriovorax* sp. DB6. *Bacteriovorax* is a genus of predatory bacteria related to *Bdellovibrio* that prey upon Gram-negative bacteria by invading their periplasmic space. They also exhibit an extracellular free-living phase in their lifecycle, during which they establish natural biofilms to deal with harsh environmental conditions (Williams et al., 2009). The 15 contigs in this group provided nearly complete genome coverage (3.73 Mb) since the average size for the four available genomes of *Bacteriovorax* spp is 3.31 Mb. However, the percentage of completeness showed that we only have 54.46% (Table S4) of the genome. The lack of members of the same genus in the databases makes the program determine marker genes only at the kingdom level. Although, the GC content of the SWde contigs (36.1%) and *Bacteriovorax* sp. DB6 genome (37.6%) are similar, ANI (72% with 48% coverage) indicated that they were clearly separate species. Phylogenetic analysis (64 conserved proteins) supported that the SWde Bacteriovorax contigs were grouped together with *Bacteriovorax* spp. cluster IX (group based on 16S rRNA, Chen et al., 2015) in the Bdellovibrionaceae branch of Deltaproteobacteria (Figure S7A). The presence of *Bacteriovorax* suggests that under specific environmental conditions the predation exhibited by this microbe could be an important factor in shaping the community composition. Although, it has been suggested that *Bacteriovorax* showed preferential predation toward *Vibrio parahaemolyticus* in seawater (Richards et al., 2013), neither assembled contigs nor raw reads obtained in the SWde metagenome could be assigned to *Vibrio*. Annotation of these genomes gave a very high rate of hypothetical proteins (annotated genes account only for ~32%) which complicates comparisons among them.

A group of Gammaproteobacteria contigs resembled the not yet formally described species *Vibrio* sp. F10 and was observed only in the SWchi metagenome. *Vibrio* sp. F10 appears to be a highly specific zooplankton saprophyte (Preheim et al., 2011). The ANI between this group of contigs (26 adding up to 2.6 Mb) and the *Vibrio* sp. F10 draft genome (77% with 53% coverage) indicated that the enriched microbe was a different species, although with a similar GC content (*ca*. 45%). The metagenomic contigs alignment to *Vibrio* sp. F10 also indicated low synteny. A phylogenetic tree using 86 conserved proteins supports the grouping of the SWchi enriched vibrio contigs with *Vibrio* sp. F10, which forms a distinct cluster from other characterized vibrios (Figure S7B). Notably, the Vibrio contigs from the SWchi metagenome contain two chitinases supporting their association with this substrate.

The chitin particles were colonized by *Vibrio* species related to the genome described as *Vibrio* sp. F10, while the cellulose particles were colonized by members of the Flavobacterium genus. Slightly more surprising, however, was the development of distinct communities associated with the particles providing no additional carbon sources. Diatomaceous earth, for example, is a material made up of the silicate remains of fossilized diatoms and provides no organic carbon, similar to sand. Nonetheless, we observed very different communities adhering to both silicate-based materials. In this case the physical structure of diatomaceous earth, which is much more convoluted and full of crevices, could be the major factor. Additional experiments with biological replicates, not available at this point, would strengthen the value of the results. Another important factor which might contribute to the differences in community composition on the different particles could be also the differences in the adsorption of DOM on these particles, depending on the surface charge and the surface structure. This is critical in primary film formation which in turn, determines the microbes colonizing the surface. Also quite remarkable was that the most fully assembled microbe in this enrichment was a predatory bacterium. These micro-predators have been isolated in different aquatic systems (marine, estuaries, and freshwater; Jurkevitch, 2007) and takes advantage of small perturbations in the environment that allow rapid growth of prey organisms (Williams et al., 2016). However, although the predatory life cycle has been well studied (Rendulic et al., 2004) little is known about the extracellular free-living phase because most of them are unable to grow in the absence of prey. It is possible that *Bacteriovorax* takes advantage of the complex surface structure to find prey more easily.

### New groups of marine bacteria

Within the classification of long contigs (>10 kb; Table 1), we found some representing unclassified microbes in which the majority of genes gave best hits to different organisms of the same class. We have used a combination of GC%, coverage, and principal component analysis of tetranucleotide frequencies in order to bin the unknown contigs into groups as was previously described (Mizuno et al., 2015). Table 2 summarizes some of the statistics of these new groups of Alphaproteobacteria, Bacteroidetes, and Gammaproteobacteria found in the PE metagenomes. Every group seems to be different in each metagenome except for the contigs belonging to Gammaproteobacteria for which the same group appeared to be present in all the metagenomes.

**Table 2:** **Summary statistics of the reconstructed genomes obtained in the enrichment metagenomes**.

**Cluster name**	**Number of contigs**	**GC (%)**	**Total length (bp)**	**Longest contig size (bp)**	**% completeness (Raes)**	**% completeness (Albertsen)**
Alphaproteobacteria MedPE-SWcel	54	61.4	4,076,435	311,167	100	83.8
Alphaproteobacteria MedPE-SWchi	100	56.6	3,548,614	120,524	80	68.5
Bacteroidetes MedPE-SWsnd-G1	60	33.4	2,702,127	196,890	85.7	75.7
Bacteroidetes MedPE-SWsnd-G2	19	34.3	3,035,083	480,119	100	83.8
Gammaproteobacteria MedPE	181	40.8	3,258,706	83,223	91.4	74.8

We have used orthologous markers to estimate the completeness of the recovered genomes. These markers indicated that the range of genome recovery was higher than 68.5% using Albertsen estimation (Albertsen et al., 2013) and 80% for Raes (Raes et al., 2007) for all the assembled genomes. Unfortunately, only in one of these groups we found a contig containing a partial 16S rRNA gene (Gammaproteobacteria MedPE). Reconstructed phylogeny of the 16S rRNA showed that our groups of contigs were related to other uncultured microbes being most similar to a bacterium associated with the sponge *Petrosia ficiformis* collected along the North Western Mediterranean coast named Gamma proteobacterium MOLA 543. In order to make a more robust phylogenetic affiliation and be able to place the groups without any 16S rRNA among the contigs we also used a concatenate of conserved proteins between these and several reference genomes chosen based on the majority of genes in the contigs giving highest similarities to genes in databases. Between the two groups of Alphaproteobacteria the SWcel group clustered together in the tree with members of the family Rhodobacteraceae (Figure S8A). Highest similarity based on ANI (77%) was with *Silicibacter* sp. strain TM1040 an isolate obtained from a culture of the dinoflagellate *Pfiesteria piscicida*. However, SWsnd contigs bin appeared as an outgroup of the tree probably forming a new genus. The assembled contigs of both new Alphaproteobacteria were submitted to the RAST annotation server for subsystem classification (Aziz et al., 2008). SWcel Alphaproteobacteria revealed an increase number in genes involved in membrane transport such as ABC transporters, iron acquisition and carbohydrates including mono, polysaccharide utilization and one-carbon metabolism in comparison to the SWsnd. However, the main difference in the Alphaproteobacteria SWsnd genome was the presence of a nitrous oxide reductase cluster of genes (*nos*RZDFYLX) involved in denitrification. Two new groups of Bacteroidetes came from the same metagenome (SWsnd), the resulting trees (Figure S8B) with several reference Bacteroidetes genomes showed that nearest neighbors for the first group were *Polaribacter* spp and *Winogradskyella* spp for the second. However, their similarity with the some representative genomes was rather low (ANI <60%), suggesting that they belong to different genera. All these five new groups are estimated to be >79% complete with minimal estimated contamination (<1.8%) (Table S4). Estimating the percentage of identical reads that match to the corresponding metagenome, these partial genomes accumulated between 3 and 9% of the reads (Table S4).

### New marine rhizobiales phage

**Figure 4:**
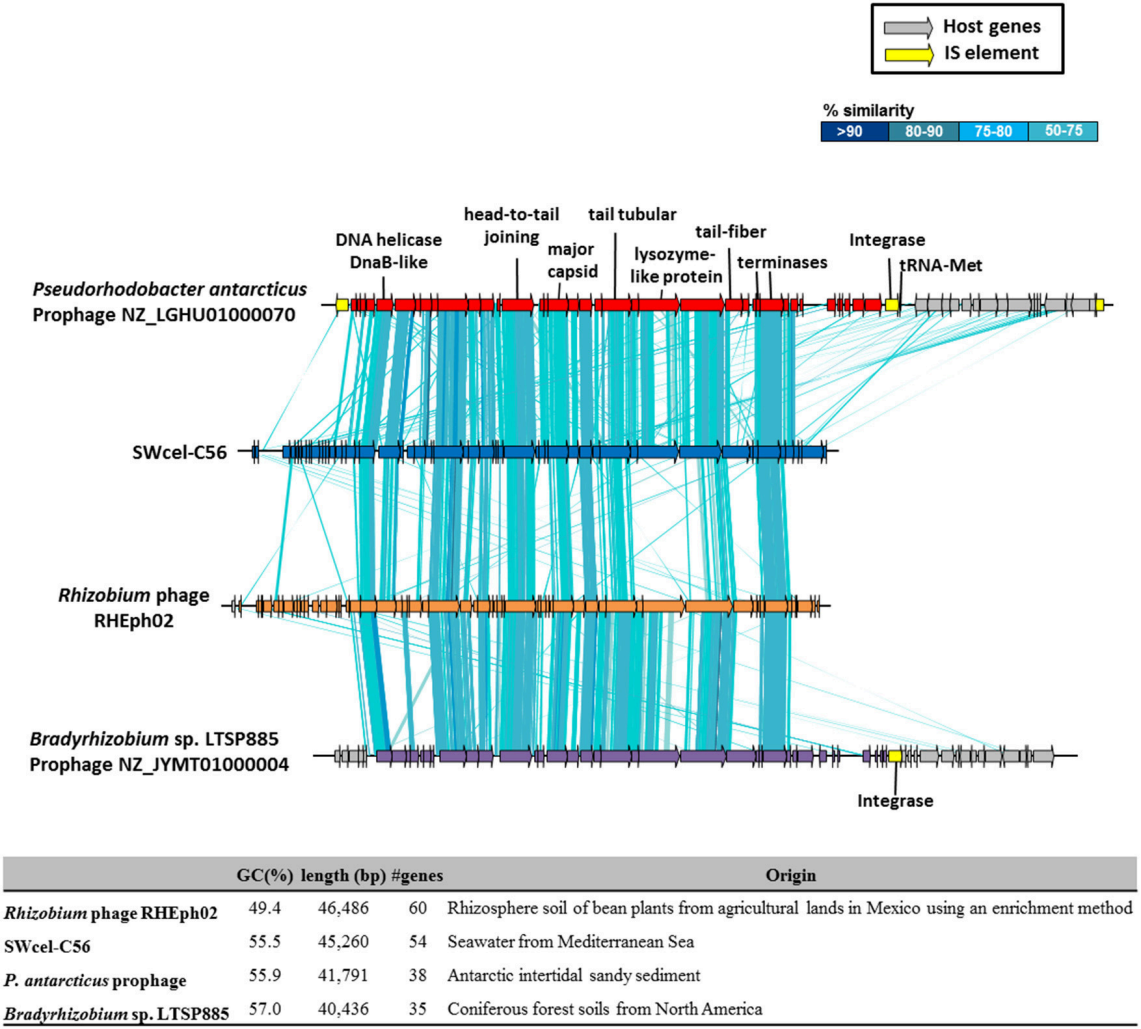
**Schematic representation and comparison of the putative phage found in the SWcel enriched metagenome and the closely related phages and prophages based on the sequence of the terminase (Figure S9)**. The table shows the general features of the genomes.

From all the assembled long contigs (>10 kb) some of them were related to phages. Most affiliated with *Vibrio* and *Pseudoalteromonas* phages based on sequence similarities. However, in the SWcel metagenome we have found a 45 Kb phage with 55.5% GC content encoding 54 CDSs, of which 18 showed similarities to a group of lytic phages that infect *Rhizobium etli* isolated from rhizosphere soil of bean plants from agricultural lands in Mexico (Santamaría et al., 2014). Despite the different origin (soil and seawater) synteny between these two phages was well conserved (Figure 4). Based on the large terminase protein of this phage, we built a phylogenetic tree (Figure S9) using the 100 most similar sequences identified in GenBank via BLASTP. The tree shows that, although the SWcel phage grouped together with the *Rhizobium* phage RHEph02 and RHEph08, it was more similar to a hypothetical protein found in *Pseudorhodobacter antarcticus*, an Alphaproteobacteria isolated from Antarctic intertidal sandy sediment (Chen et al., 2013). Alignment between the SWcel phage and *P. antarcticus* (Figure 4) revealed that this microbe had a similar and syntenic prophage inserted in a tRNA-Met. Therefore, it is remarkable that this new phage seems to belong to a group that infects a broad range of Alphaproteobacteria that live in habitats as diverse as soil, marine sediment and water column around the world, from agricultural lands in Mexico to the Mediterranean Sea through the Antarctic. Little is known about the movement of viruses among different biomes. There are evidences of some conserved phage-encoded sequences found in multiple different environments (marine, freshwater, soil, sediment, and hyper-saline environments; Breitbart et al., 2004; Short and Suttle, 2005) and experiments with viruses from soil, sediment, and freshwater that were able to infect marine microbial communities (Sano et al., 2004) suggesting that viruses are important elements of lateral gene transfer between different compartments of the biosphere.

### Recruitment of the enriched microorganisms

To examine the distribution of the novel enriched microorganisms described above we performed fragment recruitment analysis by comparing each contig group to that of numerous metagenomes from a variety of seawater samples (DeLong et al., 2006; Rusch et al., 2007; Coleman and Chisholm, 2010; Eloe et al., 2011; Quaiser et al., 2011; Smedile et al., 2013; Thompson et al., 2013; Wilkins et al., 2013; Mizuno et al., 2015; Sunagawa et al., 2015; Table S5). The resulting RPKG values, which provide an estimated coverage, revealed very low, if any, recruitment of the enriched microorganisms from natural samples. Neither the place of origin (Mediterranean Sea, Baltic Sea, Atlantic, and Pacific Ocean) nor the filter fraction used (ranging between 20 and 0.1 μm) had a significant effect in the abundance of the enriched microorganism and their most closely related genomes. Only the genomes related to *Pseudoalteromonas* species recruited significantly at some stations (TARA 70 South Atlantic Ocean [800 m] and TARA 93 from South Atlantic Ocean [5 and 30 m]) from the FL fraction in the TARA metagenomes. The lack of recruitment observed here suggest that particle enrichment, like many other culturing techniques, selects for a small subset of microorganisms that flourish in these specific conditions but do not fare so well in more complex natural environments. These kinds of microbes which exist at nominal levels under natural conditions are part of the denominated “rare biosphere” (Sogin et al., 2006). We have also analyzed the presence of all these novel microorganisms against the four PE metagenomes and the control (SW) using not only RPGK (Table S5) but also the percentage of total reads from the metagenome that match to each genome (at 100% identity; Table S4). The total percentage of metagenomic reads recruited by the genomes assembled here ranged from 17% (SWchi) to 52% (SW). Although, far from being diagnostic as to what microbes predominated in the enrichments, our results describe novel genomes that appear in significant numbers under the conditions studied.

## Conclusions

We have used two metagenomic approaches, direct sequencing of natural samples and sequencing after enrichment, to characterize communities of prokaryotes associated to particles. The direct sequencing of biomass collected on 5 μm filters, which in theory should collect eukaryotic cells and only prokaryotic cells forming aggregates or associated to particulate matter, accentuates the difficulties associated with evaluating particle-associated microbiomes. Our data has shown that “bona fide” free living bacteria, such as *Ca.* Pelagibacter, are collected in the large pore size, likely as a result of clogging that produces a much smaller pore size. In addition, the large amount of eukaryotic DNA collected complicates the analysis of these metagenomes at the level of assembly. Despite these complications, the subtractive analysis, i.e., what is present in PA that is not found in FL, indicated the existence of specific groups of Actinobacteria, Planctomycetes, Flavobacteria, and Proteobacteria that were not present in FL. These microbes appear to be undescribed as of yet, even by metagenomics, and justify the use of alternative approaches that target and retrieve this fraction of the marine microbiome.

The use of culture enrichments has provided genomic data about microbes present in the sample that attach to different particulate materials. This data revealed a remarkable degree of specificity, with totally different microbes found in the different enrichments, and very little overlap despite the fact that dissolved organic carbon provided for all the enrichments, namely pyruvate, was the same. Some of the particles provided additional sources of carbon and other nutrients that could dictate some specificity. This was particularly clear in the case of chitin where classic chitinolytic microbes of the genus Vibrio were found to dominate (Hunt et al., 2008). Inorganic material, such as sand or diatomaceous earth, however, also supported very distinct communities, suggesting that additional physical properties influence the development and survival of specific microorganisms. Although, the objective of this analysis was to identify microbes (through the reconstructed genomes) that appear when certain kinds of particles were used, in the future a more in-depth analysis should be performed including both biological replicates and microscopy to check the status of the particles at the end of the experiment. Collectively, our data reveals new and distinct bacteria from the marine microbiome and suggests that particles found in this environment likely play a role in defining the marine microbiome.

## Author contributions

FR, NK designed the project. ML, NK analyzed the data. JH contributed bioinformatics tools. ML, NK, and FR wrote the paper.

### Conflict of interest statement

The authors declare that the research was conducted in the absence of any commercial or financial relationships that could be construed as a potential conflict of interest.

## References

[B1] Albertsen M. Hugenholtz P. Skarshewski A. Nielsen K. L. Tyson G. W. Nielsen P. H. (2013). Genome sequences of rare, uncultured bacteria obtained by differential coverage binning of multiple metagenomes. Nat. Biotechnol. 31, 533–538. 10.1038/nbt.2579 23707974

[B2] Allen A. E. Allen L. Z. Mccrow J. P. (2013). Lineage specific gene family enrichment at the microscale in marine systems. Curr. Opin. Microbiol. 16, 605–617. 10.1016/j.mib.2013.10.001 24377115

[B3] Allen L. Z. Allen E. E. Badger J. H. Mccrow J. P. Paulsen I. T. Elbourne L. D.. (2012). Influence of nutrients and currents on the genomic composition of microbes across an upwelling mosaic. ISME J. 6, 1403–1414. 10.1038/ismej.2011.201 22278668 PMC3379637

[B4] Allgaier M. Grossart H.-P. (2006). Seasonal dynamics and phylogenetic diversity of free-living and particle-associated bacterial communities in four lakes in northeastern Germany. Aquat. Microbial. Ecol. 45, 115–128. 10.3354/ame045115

[B5] Altschul S. F. Madden T. L. Schäffer A. A. Zhang J. Zhang Z. Miller W.. (1997). Gapped BLAST and PSI-BLAST: a new generation of protein database search programs. Nucleic Acids Res. 25, 3389–3402. 10.1093/nar/25.17.3389 9254694 PMC146917

[B6] Azam F. (1998). Microbial control of oceanic carbon flux: the plot thickens. Science 280, 694–696. 10.1126/science.280.5364.694

[B7] Azam F. Long R. A. (2001). Oceanography: Sea snow microcosms. Nature 414, 495–498. 10.1038/35107174 11734832

[B8] Azam F. Malfatti F. (2007). Microbial structuring of marine ecosystems. Nat. Rev. Microbiol. 5, 782–791. 10.1038/nrmicro1747 17853906

[B9] Azam F. Worden A. Z. (2004). Microbes, molecules, and marine ecosystems. Science 303, 1622–1624. 10.1126/science.1093892 15016987

[B10] Aziz R. K. Bartels D. Best A. A. Dejongh M. Disz T. Edwards R. A.. (2008). The RAST Server: rapid annotations using subsystems technology. BMC Genomics 9:75. 10.1186/1471-2164-9-75 18261238 PMC2265698

[B11] Bengtsson M. M. Øvreås L. (2010). Planctomycetes dominate biofilms on surfaces of the kelp Laminaria hyperborea. BMC Microbiol. 10:261. 10.1186/1471-2180-10-261 20950420 PMC2964680

[B12] Breitbart M. Miyake J. H. Rohwer F. (2004). Global distribution of nearly identical phage-encoded DNA sequences. FEMS Microbiol. Lett. 236, 249–256. 10.1111/j.1574-6968.2004.tb09654.x 15251204

[B13] Brown S. D. Utturkar S. M. Klingeman D. M. Johnson C. M. Martin S. L. Land M. L.. (2012). Twenty-One Genome Sequences from Pseudomonas Species and 19 Genome Sequences from Diverse Bacteria Isolated from the Rhizosphere and Endosphere of Populus deltoides. J. Bacteriol. 194, 5991–5993. 10.1128/JB.01243-12 23045501 PMC3486089

[B14] Capella-Gutiérrez S. Silla-Martínez J. M. Gabaldón T. (2009). trimAl: a tool for automated alignment trimming in large-scale phylogenetic analyses. Bioinformatics 25, 1972–1973. 10.1093/bioinformatics/btp348 19505945 PMC2712344

[B15] Chen C.-X. Zhang X.-Y. Liu C. Yu Y. Liu A. Li G.-W.. (2013). Pseudorhodobacter antarcticus sp. nov., isolated from Antarctic intertidal sandy sediment, and emended description of the genus Pseudorhodobacter Uchino et al. 2002 emend. Jung et al. 2012. Int. J. Syst. Evolut. Microbiol. 63, 849–854. 10.1099/ijs.0.042184-0 22611201

[B16] Chen H. Brinkac L. M. Mishra P. Li N. Lymperopoulou D. S. Dickerson T. L.. (2015). Draft genome sequences for the obligate bacterial predators Bacteriovorax spp. of four phylogenetic clusters. Stand. Genomic Sci. 10:11. 10.1186/1944-3277-10-11 26203326 PMC4511183

[B17] Coleman M. L. Chisholm S. W. (2010). Ecosystem-specific selection pressures revealed through comparative population genomics. Proc. Natl. Acad. Sci. 107, 18634–18639. 10.1073/pnas.1009480107 20937887 PMC2972931

[B18] Crespo B. G. Pommier T. Fernández-Gómez B. Pedrós-Alió C. (2013). Taxonomic composition of the particle-attached and free-living bacterial assemblages in the Northwest Mediterranean Sea analyzed by pyrosequencing of the 16S rRNA. Microbiol. Open 2, 541–552. 10.1002/mbo3.92 23723056 PMC3948605

[B19] Crump B. C. Armbrust E. V. Baross J. A. (1999). Phylogenetic analysis of particle-attached and free-living bacterial communities in the Columbia River, its estuary, and the adjacent coastal ocean. Appl. Environ. Microbiol. 65, 3192–3204. 10388721 10.1128/aem.65.7.3192-3204.1999 PMC91474

[B20] DeLong E. (2013). Microbial Metagenomics, Metatranscriptomics, and Metaproteomics. San Diego, CA: Academic Press. 10.1016/B978-0-12-407863-5.09983-4 24060136

[B21] DeLong E. F. Franks D. G. Alldredge A. L. (1993). Phylogenetic diversity of aggregate-attached vs. free-living marine bacterial assemblages. Limnol. Oceanogr. 38, 924–934. 10.4319/lo.1993.38.5.0924

[B22] DeLong E. F. Preston C. M. Mincer T. Rich V. Hallam S. J. Frigaard N.-U.. (2006). Community genomics among stratified microbial assemblages in the ocean's interior. Science 311, 496–503. 10.1126/science.1120250 16439655

[B23] Deng W. Monks L. Neuer S. (2015). Effects of clay minerals on the aggregation and subsequent settling of marine *Synechococcus*. Limnol. Oceanogr. 60, 805–816. 10.1002/lno.10059

[B24] Dinsdale E. A. Edwards R. A. Hall D. Angly F. Breitbart M. Brulc J. M.. (2008). Functional metagenomic profiling of nine biomes. Nature 452, 629–632. 10.1038/nature06810 18337718

[B25] Dupont C. L. Mccrow J. P. Valas R. Moustafa A. Walworth N. Goodenough U.. (2015). Genomes and gene expression across light and productivity gradients in eastern subtropical Pacific microbial communities. ISME J. 9, 1076–1092. 10.1038/ismej.2014.198 25333462 PMC4410273

[B26] Edgar R. C. (2010). Search and clustering orders of magnitude faster than BLAST. Bioinformatics 26, 2460–2461. 10.1093/bioinformatics/btq461 20709691

[B27] Eloe E. A. Fadrosh D. W. Novotny M. Zeigler Allen L. Kim M. Lombardo M.-J.. (2011). Going deeper: metagenome of a hadopelagic microbial community. PLoS ONE 6:e20388. 10.1371/journal.pone.0020388 21629664 PMC3101246

[B28] Fierer N. Leff J. W. Adams B. J. Nielsen U. N. Bates S. T. Lauber C. L.. (2012). Cross-biome metagenomic analyses of soil microbial communities and their functional attributes. Proc. Natl. Acad. Sci. U.S.A. 109, 21390–21395. 10.1073/pnas.1215210110 23236140 PMC3535587

[B29] Finn R. D. Clements J. Eddy S. R. (2011). HMMER web server: interactive sequence similarity searching. Nucleic Acids Res. 39, W29–W37. 10.1093/nar/gkr367 21593126 PMC3125773

[B30] Fontanez K. M. Eppley J. M. Samo T. J. Karl D. M. Delong E. F. (2015). Microbial community structure and function on sinking particles in the North Pacific Subtropical Gyre. Front. Microbiol. 6, 469. 10.3389/fmicb.2015.00469 26042105 PMC4436931

[B31] Fu Y. Tang X. Lai Q. Zhang C. Zhong H. Li W.. (2011). *Flavobacterium beibuense* sp. nov., isolated from marine sediment. Int. J. Syst. Evolut. Microbiol. 61, 205–209. 10.1099/ijs.0.018846-0 20190020

[B32] Ganesh S. Parris D. J. Delong E. F. Stewart F. J. (2014). Metagenomic analysis of size-fractionated picoplankton in a marine oxygen minimum zone. ISME J. 8, 187–211. 10.1038/ismej.2013.144 24030599 PMC3869020

[B33] Ghai R. Martin-Cuadrado A.-B. Molto A. G. Heredia I. G. Cabrera R. Martin J.. (2010). Metagenome of the Mediterranean deep chlorophyll maximum studied by direct and fosmid library 454 pyrosequencing. ISME J. 4, 1154–1166. 10.1038/ismej.2010.44 20393571

[B34] Ghai R. Mizuno C. M. Picazo A. Camacho A. Rodriguez-Valera F. (2013). Metagenomics uncovers a new group of low GC and ultra-small marine Actinobacteria. Sci. Rep. 3:2471. 10.1038/srep02471 23959135 PMC3747508

[B35] Giovannoni S. J. Tripp H. J. Givan S. Podar M. Vergin K. L. Baptista D.. (2005). Genome streamlining in a cosmopolitan oceanic bacterium. Science 309, 1242–1245. 10.1126/science.1114057 16109880

[B36] Gray J. P. Herwig R. P. (1996). Phylogenetic analysis of the bacterial communities in marine sediments. Appl. Environ. Microbiol. 62, 4049–4059. 8899989 10.1128/aem.62.11.4049-4059.1996 PMC168226

[B37] Haft D. H. Loftus B. J. Richardson D. L. Yang F. Eisen J. A. Paulsen I. T.. (2001). TIGRFAMs: a protein family resource for the functional identification of proteins. Nucleic Acids Res. 29, 41–43. 10.1093/nar/29.1.41 11125044 PMC29844

[B38] Huang Y. Gilna P. Li W. (2009). Identification of ribosomal RNA genes in metagenomic fragments. Bioinformatics 25, 1338–1340. 10.1093/bioinformatics/btp161 19346323 PMC2677747

[B39] Hunt D. E. Gevers D. Vahora N. M. Polz M. F. (2008). Conservation of the chitin utilization pathway in the Vibrionaceae. Appl. Environ. Microbiol. 74, 44–51. 10.1128/AEM.01412-07 17933912 PMC2223224

[B40] Hyatt D. Chen G.-L. Locascio P. F. Land M. L. Larimer F. W. Hauser L. J. (2010). Prodigal: prokaryotic gene recognition and translation initiation site identification. BMC Bioinformatics 11:119. 10.1186/1471-2105-11-119 20211023 PMC2848648

[B41] Jiao N. Azam F. (2011). Microbial carbon pump and its significance for carbon sequestration in the ocean, in Microbial Carbon Pump in the Ocean, Vol. 10, eds Jiao N. Azam F. Sanders S. (Washington, DC: Science/AAAS), 43–45.

[B42] Jurkevitch E. (2007). Predatory behaviors in bacteria-diversity and transitions. Microbe Am. Soc. Microbiol. 2:67 10.1128/microbe.2.67.1

[B43] Kimes N. E. López-Pérez M. Flores-Félix J. D. Ramírez-Bahena M.-H. Igual J. M. Peix A.. (2015). Pseudorhizobium pelagicum gen. nov., sp. nov. isolated from a pelagic Mediterranean zone. Syst. Appl. Microbiol. 38, 293–299. 10.1016/j.syapm.2015.05.003 26078205

[B44] Kiørboe T. Jackson G. A. (2001). Marine snow, organic solute plumes, and optimal chemosensory behavior of bacteria. Limnol. Oceanogr. 46, 1309–1318. 10.4319/lo.2001.46.6.1309

[B45] Klippel B. Lochner A. Bruce D. C. Davenport K. W. Detter C. Goodwin L. A.. (2011). Complete genome sequences of krokinobactersp. Strain 4H-3-7-5 and Lacinutrixsp. Strain 5H-3-7-4, Polysaccharide-degrading members of the family Flavobacteriaceae. J. Bacteriol. 193, 4545–4546. 10.1128/JB.05518-11 21725025 PMC3165524

[B46] Lassmann T. Sonnhammer E. L. (2005). Kalign–an accurate and fast multiple sequence alignment algorithm. BMC Bioinformat. 6:298. 10.1186/1471-2105-6-298 16343337 PMC1325270

[B47] Lavigne H. D'ortenzio F. Ribera D'alcalà M. Claustre H. Sauzède R. Gacic M. (2015). On the vertical distribution of the chlorophyll a concentration in the Mediterranean Sea: a basin scale and seasonal approach. Biogeosci. Discuss. 12, 4139–4181. 10.5194/bgd-12-4139-2015

[B48] Longford S. R. Tujula N. A. Crocetti G. R. Holmes A. J. Holmström C. Kjelleberg S. (2007). Comparisons of diversity of bacterial communities associated with three sessile marine eukaryotes. Aquat. Microbial. Ecol. 48, 217–229. 10.3354/ame048217

[B49] Lowe T. M. Eddy S. R. (1997). tRNAscan-SE: a program for improved detection of transfer RNA genes in genomic sequence. Nucleic Acids Res. 25, 955–964. 10.1093/nar/25.5.0955 9023104 PMC146525

[B50] Mackelprang R. Waldrop M. P. Deangelis K. M. David M. M. Chavarria K. L. Blazewicz S. J.. (2011). Metagenomic analysis of a permafrost microbial community reveals a rapid response to thaw. Nature 480, 368–371. 10.1038/nature10576 22056985

[B51] Mizuno C. M. Rodriguez-Valera F. Ghai R. (2015). Genomes of Planktonic Acidimicrobiales: Widening Horizons for Marine Actinobacteria by Metagenomics. MBio 6, e02083–e02014. 10.1128/mBio.02083-14 25670777 PMC4337565

[B52] Mizuno C. M. Rodriguez-Valera F. Kimes N. E. Ghai R. (2013). Expanding the marine virosphere using metagenomics. PLoS Genet. 9:e1003987. 10.1371/journal.pgen.1003987 24348267 PMC3861242

[B53] Nawrocki E. P. (2009). Structural RNA Homology Search and Alignment Using Covariance Models. Ph.D. thesis, Washington University School of Medicine, St. Louis, USA.

[B54] Parks D. H. Imelfort M. Skennerton C. T. Hugenholtz P. Tyson G. W. (2015). CheckM: assessing the quality of microbial genomes recovered from isolates, single cells, and metagenomes. Genome Res. 25, 1043–1055. 10.1101/gr.186072.114 25977477 PMC4484387

[B55] Peng Y. Leung H. C. Yiu S.-M. Chin F. Y. (2012). IDBA-UD: a de novo assembler for single-cell and metagenomic sequencing data with highly uneven depth. Bioinformatics 28, 1420–1428. 10.1093/bioinformatics/bts174 22495754

[B56] Preheim S. P. Boucher Y. Wildschutte H. David L. A. Veneziano D. Alm E. J.. (2011). Metapopulation structure of Vibrionaceae among coastal marine invertebrates. Environ. Microbiol. 13, 265–275. 10.1111/j.1462-2920.2010.02328.x 20819104

[B57] Price M. N. Dehal P. S. Arkin A. P. (2010). FastTree 2–approximately maximum-likelihood trees for large alignments. PLoS ONE 5:e9490. 10.1371/journal.pone.0009490 20224823 PMC2835736

[B58] Qin J. Li R. Raes J. Arumugam M. Burgdorf K. S. Manichanh C.. (2010). A human gut microbial gene catalogue established by metagenomic sequencing. Nature 464, 59–65. 10.1038/nature08821 20203603 PMC3779803

[B59] Quaiser A. Zivanovic Y. Moreira D. López-García P. (2011). Comparative metagenomics of bathypelagic plankton and bottom sediment from the Sea of Marmara. ISME J. 5, 285–304. 10.1038/ismej.2010.113 20668488 PMC3105693

[B60] Raes J. Korbel J. O. Lercher M. J. Von Mering C. Bork P. (2007). Prediction of effective genome size in metagenomic samples. Genome Biol. 8:R10. 10.1186/gb-2007-8-1-r10 17224063 PMC1839125

[B61] Rendulic S. Jagtap P. Rosinus A. Eppinger M. Baar C. Lanz C.. (2004). A predator unmasked: life cycle of Bdellovibrio bacteriovorus from a genomic perspective. Science 303, 689–692. 10.1126/science.1093027 14752164

[B62] Richards G. P. Watson M. A. Boyd E. F. Burkhardt W. Lau R. Uknalis J.. (2013). Seasonal levels of the Vibrio predator bacteriovorax in atlantic, pacific, and gulf coast seawater. Int. J. Microbiol. 2013:375371. 10.1155/2013/375371 24454382 PMC3881529

[B63] Rieck A. Herlemann D. P. Jürgens K. Grossart H.-P. (2015). Particle-associated differ from free-living Bacteria in surface waters of the Baltic Sea. Front. Microbiol. 6. 10.3389/fmicb.2015.01297 26648911 PMC4664634

[B64] Rocap G. Larimer F. W. Lamerdin J. Malfatti S. Chain P. Ahlgren N. A.. (2003). Genome divergence in two Prochlorococcus ecotypes reflects oceanic niche differentiation. Nature 424, 1042–1047. 10.1038/nature01947 12917642

[B65] Rusch D. B. Halpern A. L. Sutton G. Heidelberg K. B. Williamson S. Yooseph S.. (2007). The Sorcerer II global ocean sampling expedition: northwest Atlantic through eastern tropical Pacific. PLoS Biol. 5:e77. 10.1371/journal.pbio.0050077 17355176 PMC1821060

[B66] Sano E. Carlson S. Wegley L. Rohwer F. (2004). Movement of viruses between biomes. Appl. Environ. Microbiol. 70, 5842–5846. 10.1128/AEM.70.10.5842-5846.2004 15466522 PMC522096

[B67] Santamaría R. I. Bustos P. Sepúlveda-Robles O. Lozano L. Rodríguez C. Fernández J. L.. (2014). Narrow-host-range bacteriophages that infect Rhizobium etli associate with distinct genomic types. Appl. Environ. Microbiol. 80, 446–454. 10.1128/AEM.02256-13 24185856 PMC3911081

[B68] Satinsky B. M. Crump B. C. Smith C. B. Sharma S. Zielinski B. L. Doherty M.. (2014). Microspatial gene expression patterns in the Amazon River Plume. Proc. Natl. Acad. Sci. 111, 11085–11090. 10.1073/pnas.1402782111 25024226 PMC4121788

[B69] Segata N. Waldron L. Ballarini A. Narasimhan V. Jousson O. Huttenhower C. (2012). Metagenomic microbial community profiling using unique clade-specific marker genes. Nat. Methods 9, 811–814. 10.1038/nmeth.2066 22688413 PMC3443552

[B70] Short C. M. Suttle C. A. (2005). Nearly identical bacteriophage structural gene sequences are widely distributed in both marine and freshwater environments. Appl. Environ. Microbiol. 71, 480–486. 10.1128/AEM.71.1.480-486.2005 15640224 PMC544240

[B71] Smedile F. Messina E. La Cono V. Tsoy O. Monticelli L. S. Borghini M.. (2013). Metagenomic analysis of hadopelagic microbial assemblages thriving at the deepest part of Mediterranean Sea, Matapan-Vavilov Deep. Environ. Microbiol. 15, 167–182. 10.1111/j.1462-2920.2012.02827.x 22827264

[B72] Smith M. W. Allen L. Z. Allen A. E. Herfort L. Simon H. M. (2013). Contrasting genomic properties of free-living and particle-attached microbial assemblages within a coastal ecosystem. Front. Microbiol. 4:120. 10.3389/fmicb.2013.00120 23750156 PMC3668451

[B73] Sogin M. L. Morrison H. G. Huber J. A. Welch D. M. Huse S. M. Neal P. R.. (2006). Microbial diversity in the deep sea and the underexplored “rare biosphere”. Proc. Natl. Acad. Sci. 103, 12115–12120. 10.1073/pnas.0605127103 16880384 PMC1524930

[B74] Sunagawa S. Coelho L. P. Chaffron S. Kultima J. R. Labadie K. Salazar G.. (2015). Structure and function of the global ocean microbiome. Science 348:1261359. 10.1126/science.1261359 25999513

[B75] Tatusov R. L. Natale D. A. Garkavtsev I. V. Tatusova T. A. Shankavaram U. T. Rao B. S.. (2001). The COG database: new developments in phylogenetic classification of proteins from complete genomes. Nucleic Acids Res. 29, 22–28. 10.1093/nar/29.1.22 11125040 PMC29819

[B76] Temperton B. Giovannoni S. J. (2012). Metagenomics: microbial diversity through a scratched lens. Curr. Opin. Microbiol. 15, 605–612. 10.1016/j.mib.2012.07.001 22831844

[B77] Thompson L. R. Field C. Romanuk T. Ngugi D. Siam R. Dorry H.. (2013). Patterns of ecological specialization among microbial populations in the Red Sea and diverse oligotrophic marine environments. Ecol. Evol. 3, 1780–1797. 10.1002/ece3.593 23789085 PMC3686209

[B78] Wilkins D. Yau S. Williams T. J. Allen M. A. Brown M. V. Demaere M. Z.. (2013). Key microbial drivers in Antarctic aquatic environments. FEMS Microbiol. Rev. 37, 303–335. 10.1111/1574-6976.12007 23062173

[B79] Williams H. N. Lymperopoulou D. S. Athar R. Chauhan A. Dickerson T. L. Chen H.. (2016). Halobacteriovorax, an underestimated predator on bacteria: potential impact relative to viruses on bacterial mortality. ISME J. 10, 491–499. 10.1038/ismej.2015.129 26251870 PMC4737939

[B80] Williams H. N. Turng B.-F. Kelley J. I. (2009). Survival response of Bacteriovorax in surface biofilm versus suspension when stressed by extremes in environmental conditions. Microb. Ecol. 58, 474–484. 10.1007/s00248-009-9499-7 19267151

