# Supplementary figures and images for: Not All Particles Are Equal: The Selective Enrichment of Particle-Associated Bacteria from the Mediterranean Sea
Source: Front Microbiol. 2016 Jun 22;7:996. doi: 10.3389/fmicb.2016.00996 (PMC4916215; doi:10.3389/fmicb.2016.00996)

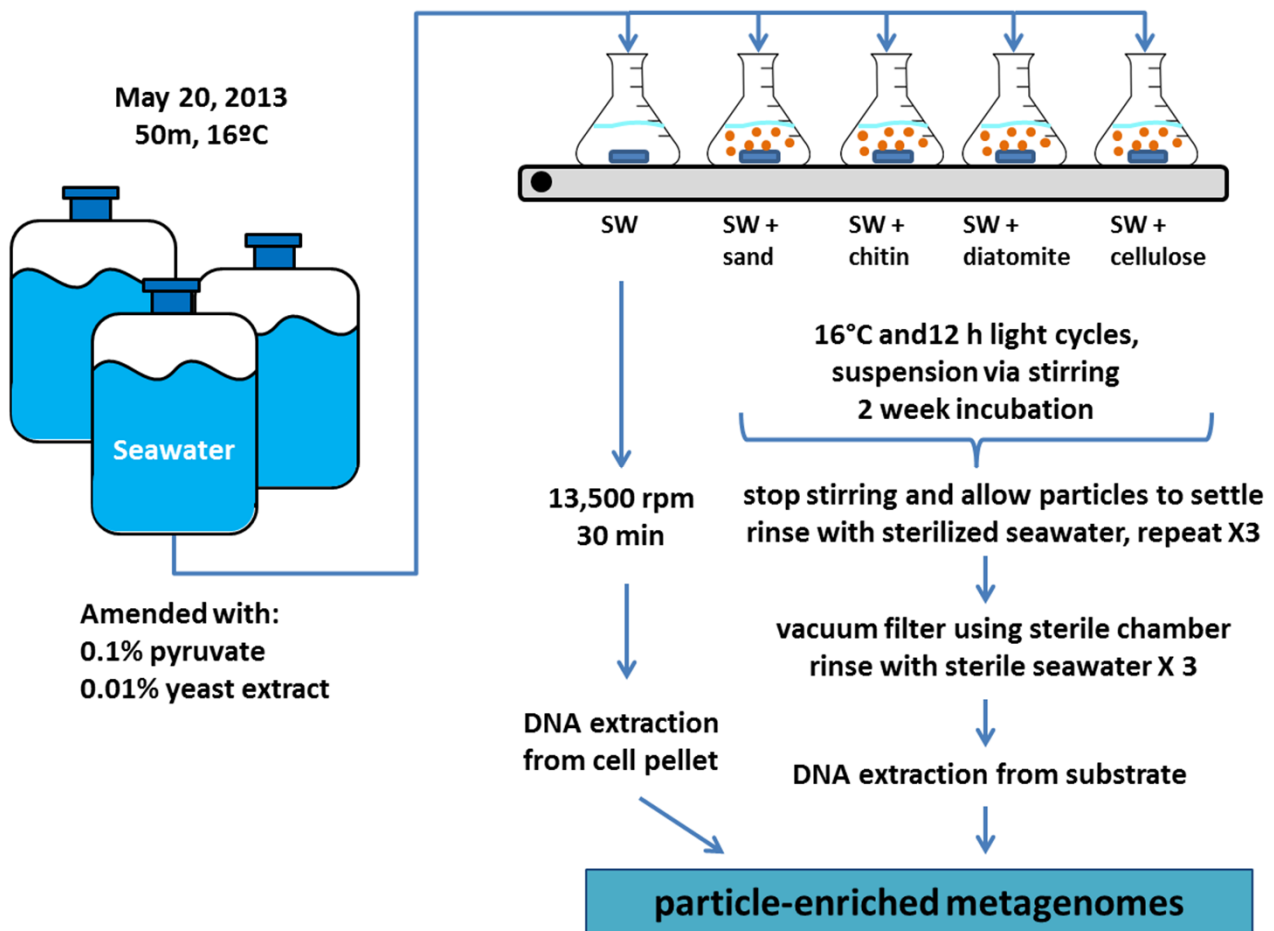

**Figure S1.** A schematic representation of the enrichments experimental design

Supplement: Supplementary file 6 [file Image1.PDF]

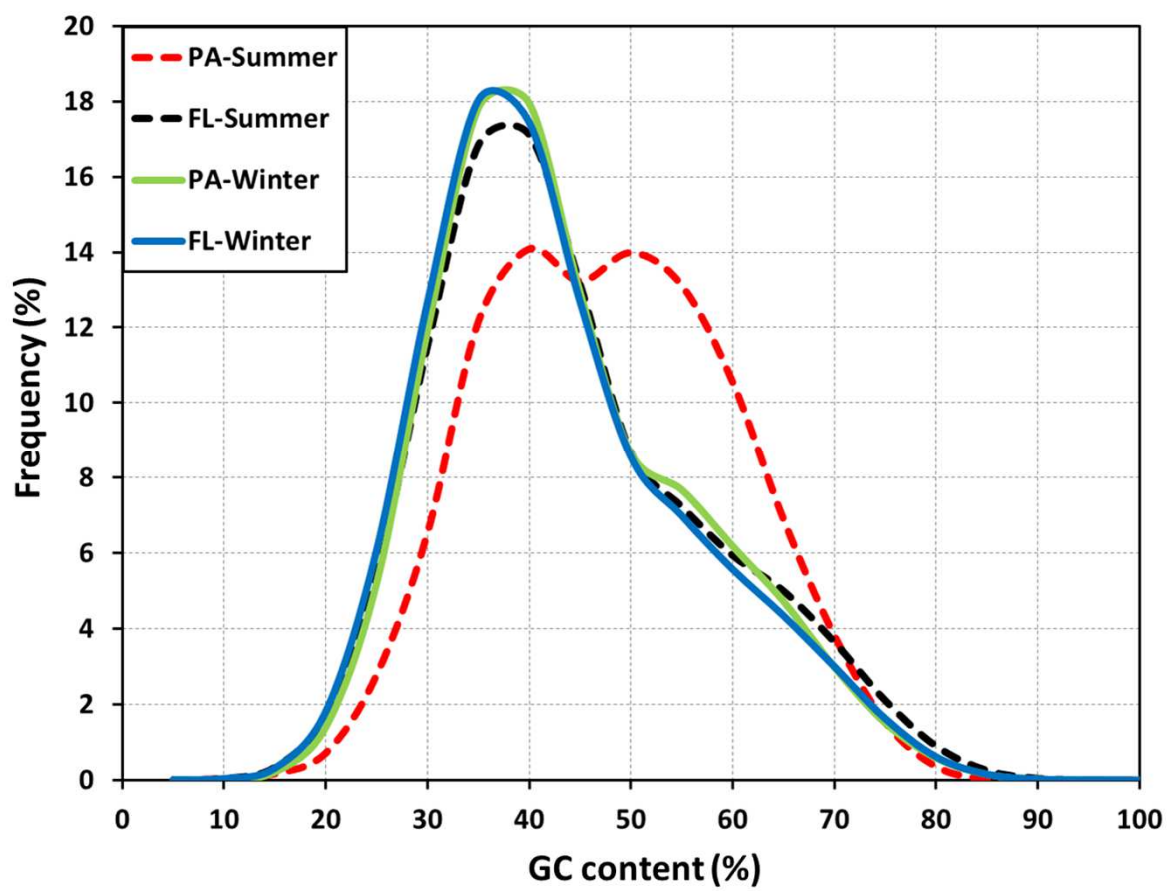

**Figure S2.-** GC content of the metagenomic datasets. Free-living (FL) and particle-associated (PA)

Supplement: Supplementary file 7 [file Image2.PDF]

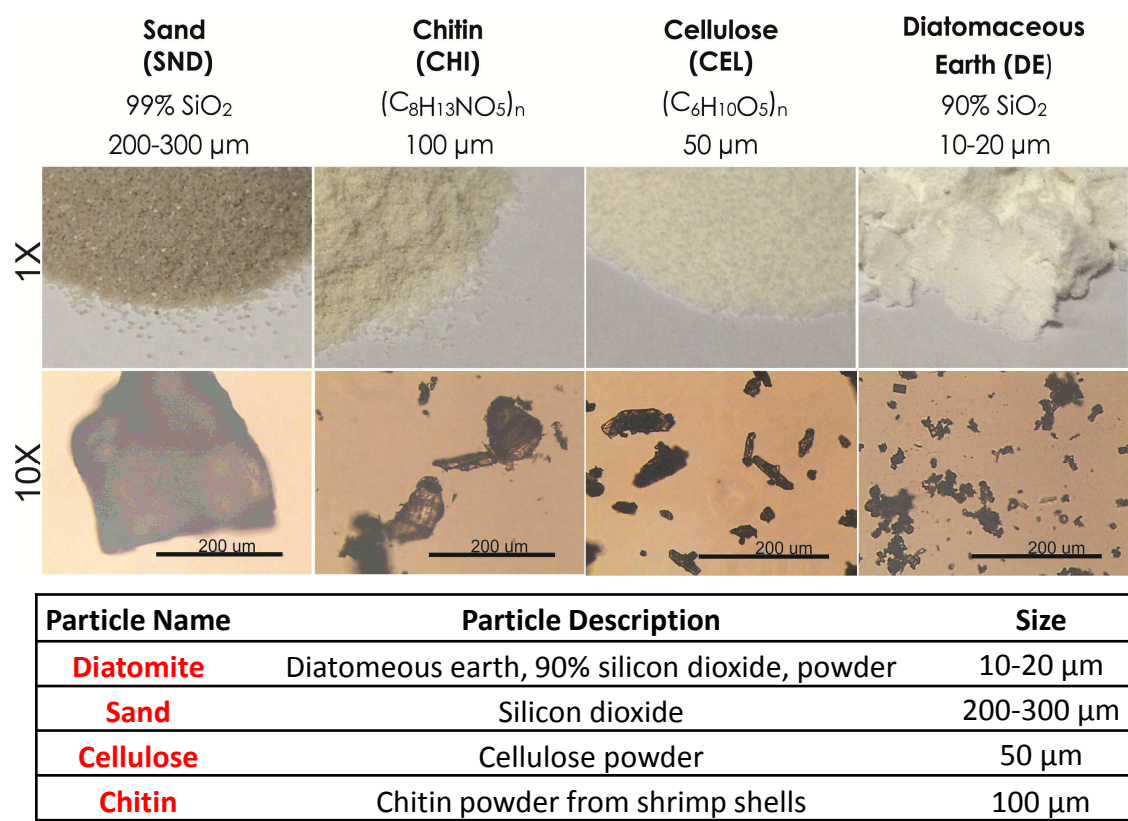

**Figure S4.** Description of particles used in this study

Supplement: Supplementary file 9 [file Image4.PDF]
