# Supplementary material for: Not All Particles Are Equal: The Selective Enrichment of Particle-Associated Bacteria from the Mediterranean Sea
Source: Front Microbiol. 2016 Jun 22;7:996. doi: 10.3389/fmicb.2016.00996 (PMC4916215; doi:10.3389/fmicb.2016.00996)
Supplement: Supplementary file 8 [file Image3.pdf]

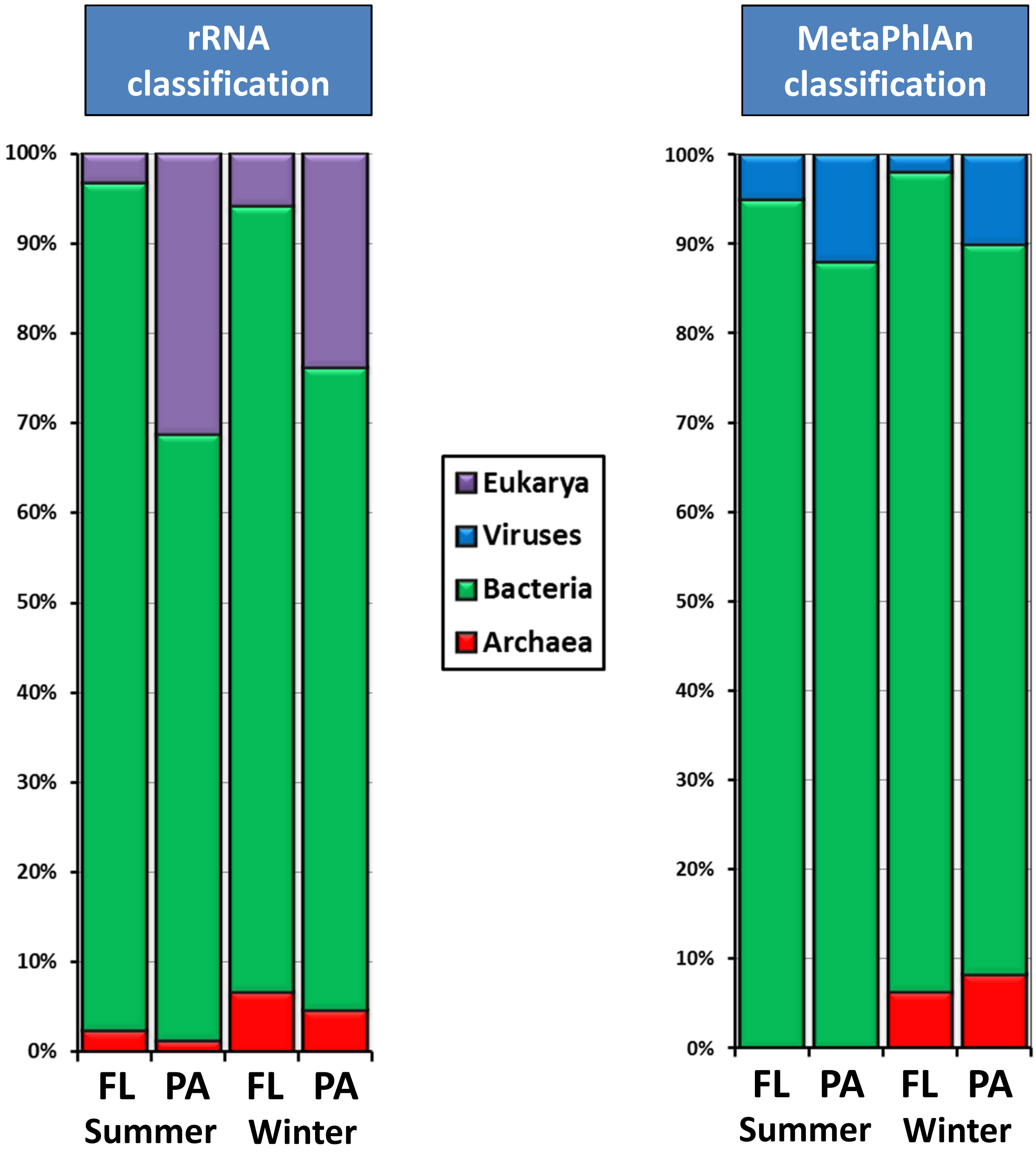

Figure S3.- Domain-level Phylogenetic classification of the metagenomes based on a) rRNA and b) MetaPhlAn from raw reads.
