# Supplementary material for: Not All Particles Are Equal: The Selective Enrichment of Particle-Associated Bacteria from the Mediterranean Sea
Source: Front Microbiol. 2016 Jun 22;7:996. doi: 10.3389/fmicb.2016.00996 (PMC4916215; doi:10.3389/fmicb.2016.00996)
Supplement: Supplementary file 10 [file Image5.PDF]

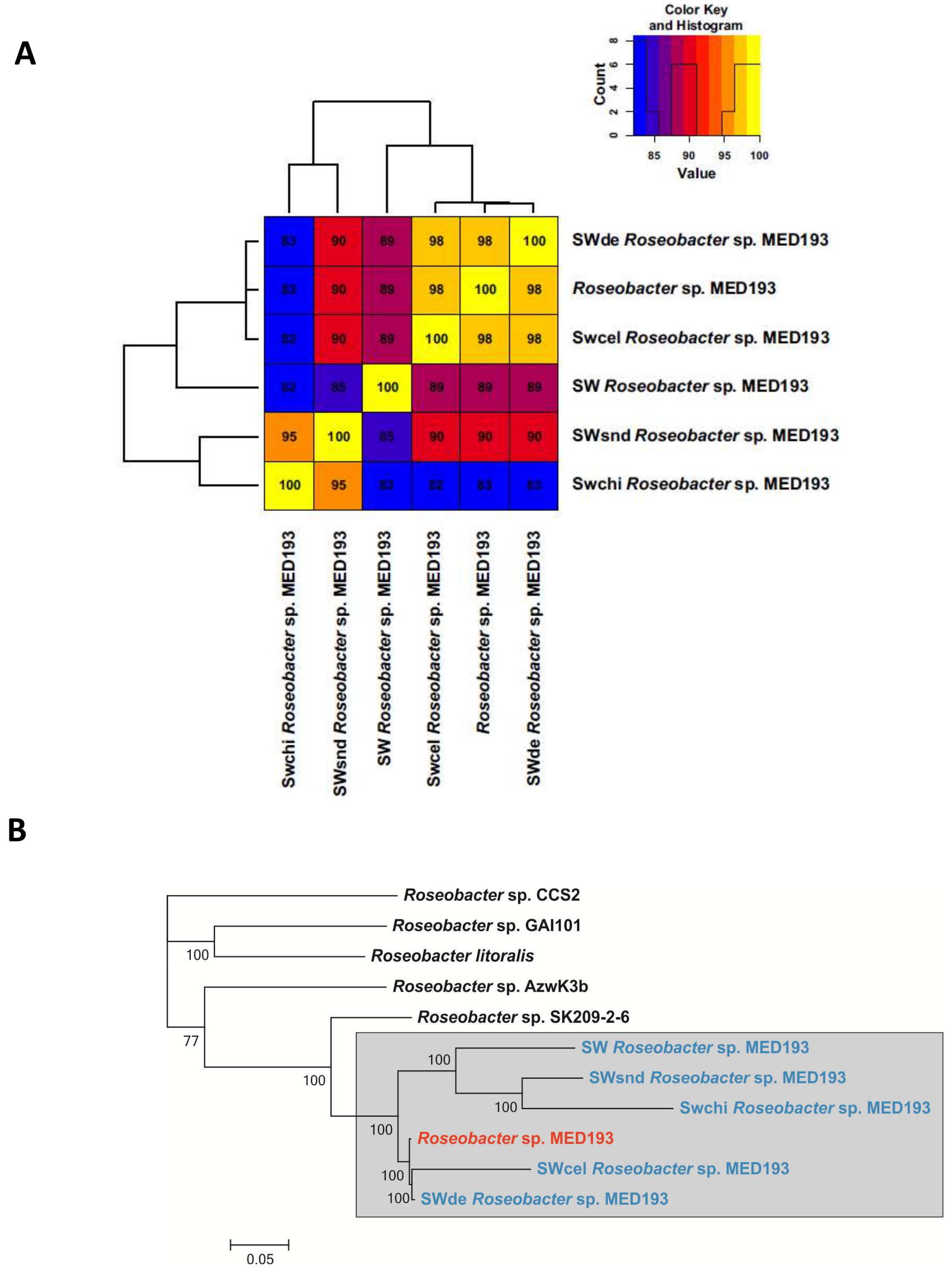

Figure S5. A) Phylogenetic analysis of the enriched Rhodobacteraceae groups. A maximum likelihood genome tree was constructed with 100 bootstraps using 42 conserved proteins among some related species and contig groups compared. B) Average Nucleotide Identity (ANI) of the genome pairwise comparisons.
