# Supplementary material for: Not All Particles Are Equal: The Selective Enrichment of Particle-Associated Bacteria from the Mediterranean Sea
Source: Front Microbiol. 2016 Jun 22;7:996. doi: 10.3389/fmicb.2016.00996 (PMC4916215; doi:10.3389/fmicb.2016.00996)
Supplement: Supplementary file 11 [file Image6.PDF]

A

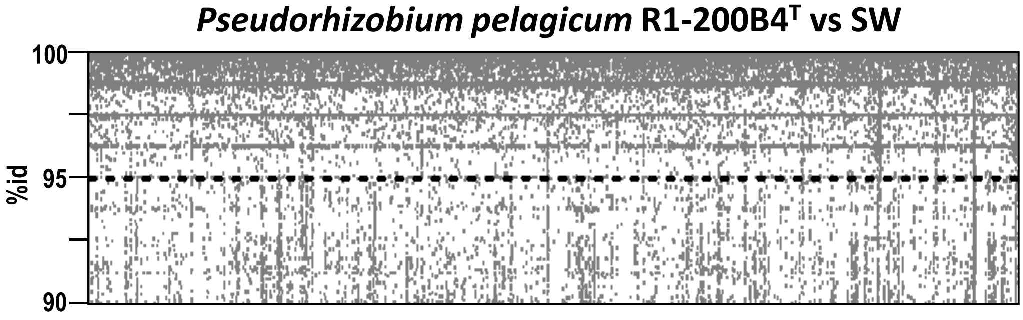

B

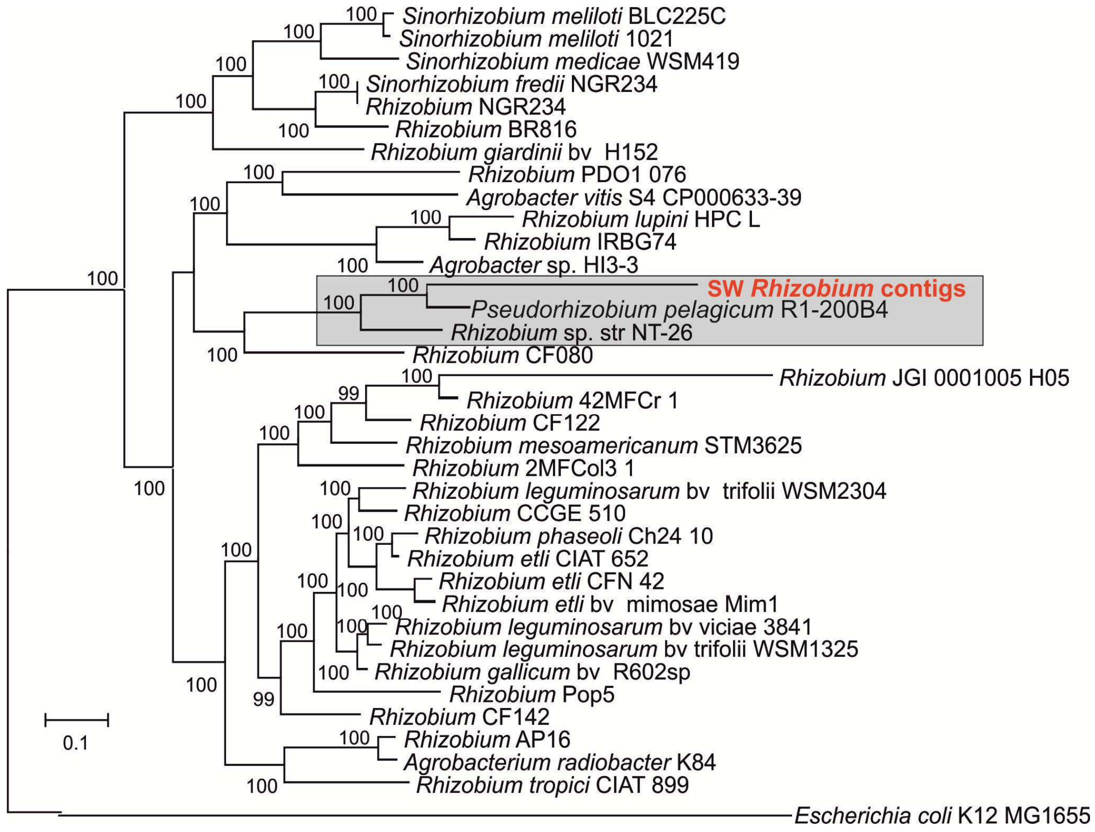

Supplementary Figure 6. The SW *Rhizobium* contigs are most closely related to a newly isolated *Pseudorhizobium pelagicum* from the Mediterranean Sea. A) Recruitment of the *Pseudorhizobium pelagicum* R1-200B4^{T} from the SW. C) Phylogenetic analysis of the SW *Rhizobium* contigs within the Rhizobium group. A maximum likelihood genome tree was constructed with 100 bootstraps using 20 conserved proteins among the 37 genomes compared.
