# Supplementary material for: Not All Particles Are Equal: The Selective Enrichment of Particle-Associated Bacteria from the Mediterranean Sea
Source: Front Microbiol. 2016 Jun 22;7:996. doi: 10.3389/fmicb.2016.00996 (PMC4916215; doi:10.3389/fmicb.2016.00996)
Supplement: Supplementary file 12 [file Image7.PDF]

A

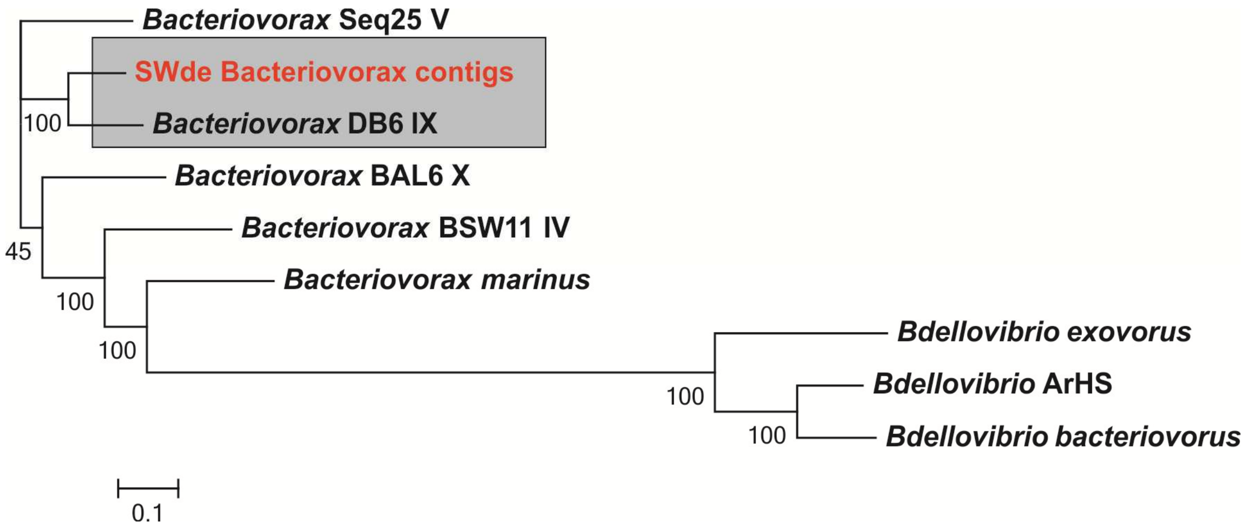

B

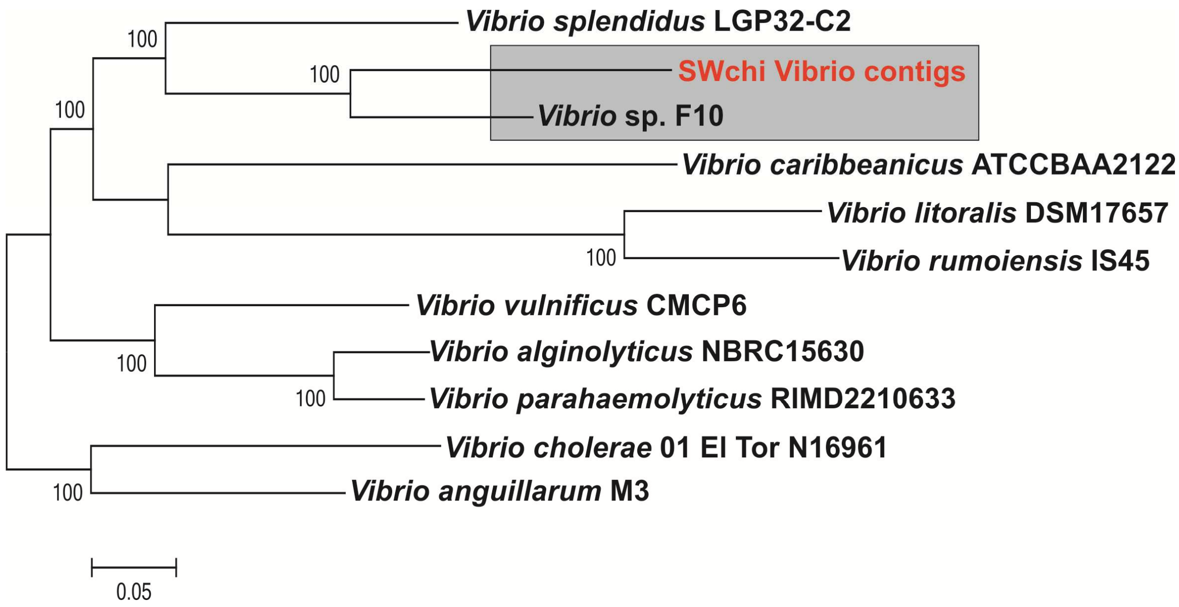

Figure S7. A) Phylogenetic analysis of the SWde enriched Bactiovorax contigs. A maximum likelihood genome tree was constructed with 100 bootstraps using 67 conserved proteins among the 8 genome and contig groups compared. B) Phylogenetic analysis of the SWchi Vibrio contigs within the Vibrio group. A maximum likelihood genome tree was constructed with 100 bootstraps using 86 conserved proteins among the 10 genomes compared.
