# Supplementary material for: Not All Particles Are Equal: The Selective Enrichment of Particle-Associated Bacteria from the Mediterranean Sea
Source: Front Microbiol. 2016 Jun 22;7:996. doi: 10.3389/fmicb.2016.00996 (PMC4916215; doi:10.3389/fmicb.2016.00996)
Supplement: Supplementary file 13 [file Image8.PDF]

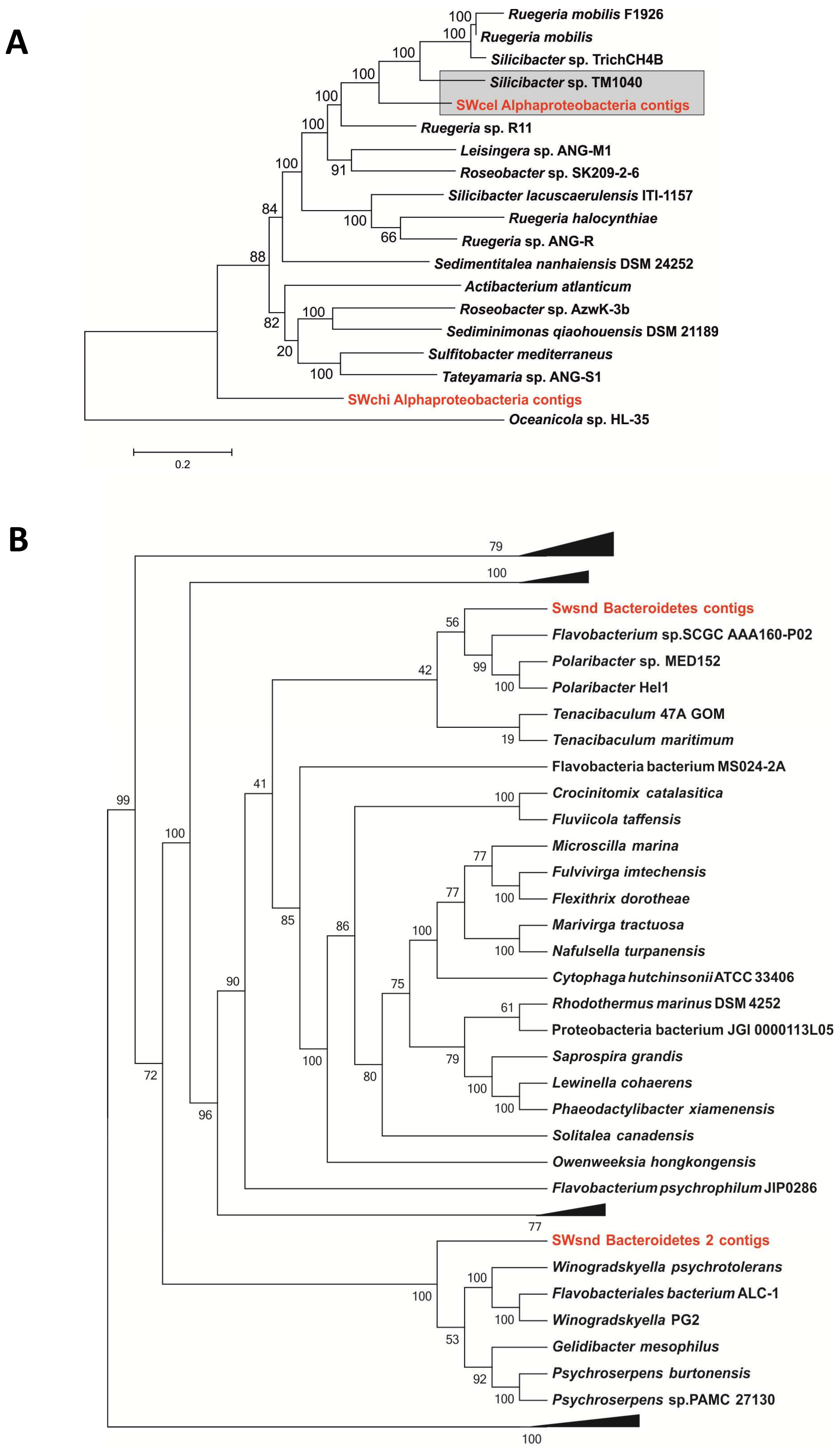

Figure S8. A) Phylogenetic analysis of the unclassified SWcel and Swchi Alphaproteobacteria contigs. A maximum likelihood genome tree was constructed with 100 bootstraps using 74 conserved proteins among the 17 genome and contig groups compared. B) Phylogenetic analysis of the two groups of SWsnd Bacteroidetes contigs. A maximum likelihood genome tree was constructed with 100 bootstraps using 45 conserved proteins among the 60 genomes compared.
