# Supplementary material for: Not All Particles Are Equal: The Selective Enrichment of Particle-Associated Bacteria from the Mediterranean Sea
Source: Front Microbiol. 2016 Jun 22;7:996. doi: 10.3389/fmicb.2016.00996 (PMC4916215; doi:10.3389/fmicb.2016.00996)
Supplement: Supplementary file 14 [file Image9.PDF]

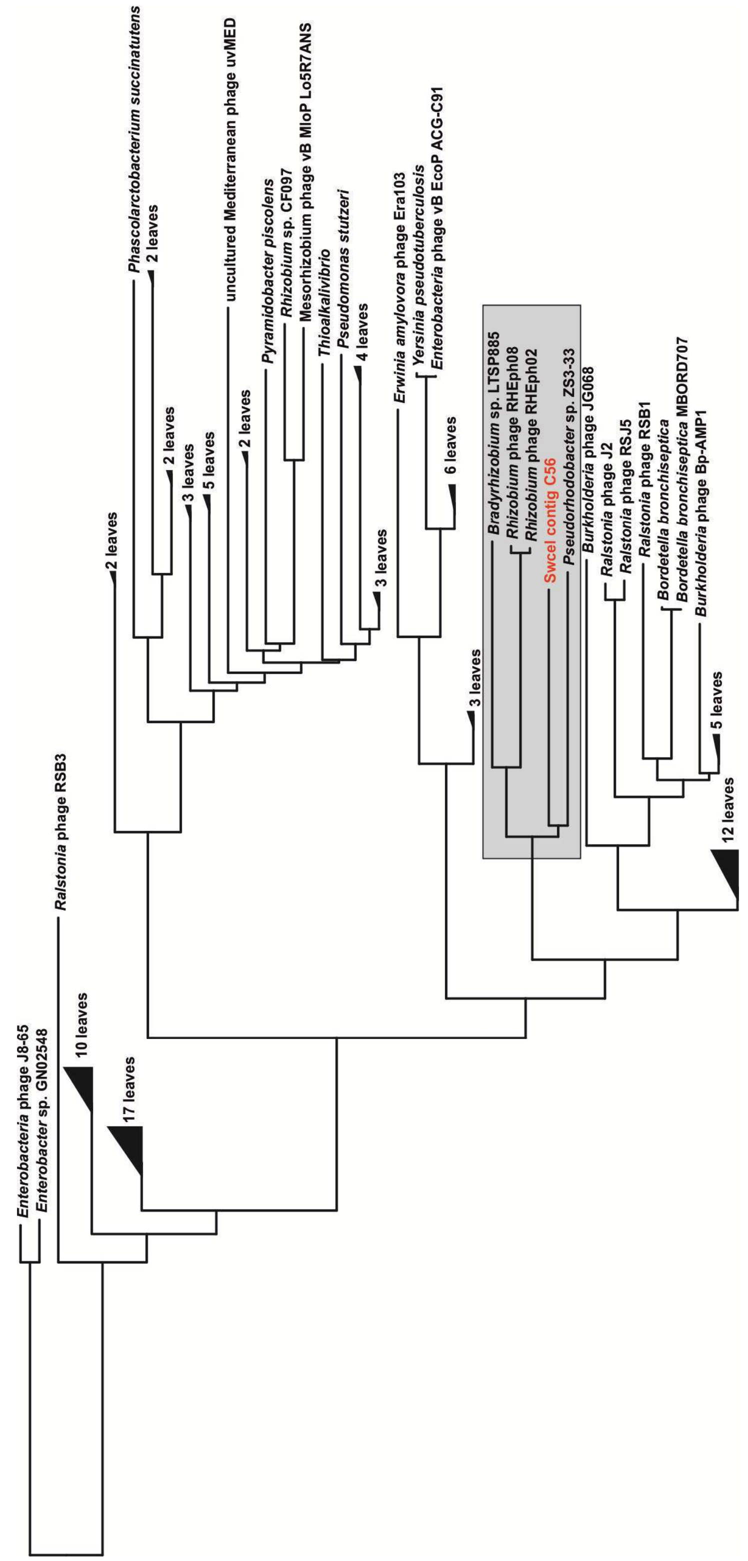

Figure S9. Tree based on the aligned amino acid sequences of the large terminase subunit with the 100 sequences more similar found in GenBank.
